# Genetic Dissection of Aversive Associative Olfactory Learning and Memory in *Drosophila* Larvae

**DOI:** 10.1371/journal.pgen.1006378

**Published:** 2016-10-21

**Authors:** Annekathrin Widmann, Marc Artinger, Lukas Biesinger, Kathrin Boepple, Christina Peters, Jana Schlechter, Mareike Selcho, Andreas S. Thum

**Affiliations:** 1 Department of Biology, University of Konstanz, Germany; 2 Zukunftskolleg, University of Konstanz, Germany; 3 Department of Neurobiology and Genetics, Theodor-Boveri-Institute, Biocenter, University of Würzburg, Germany; CNRS—ESPCI ParisTech, FRANCE

## Abstract

Memory formation is a highly complex and dynamic process. It consists of different phases, which depend on various neuronal and molecular mechanisms. In adult *Drosophila* it was shown that memory formation after aversive Pavlovian conditioning includes—besides other forms—a labile short-term component that consolidates within hours to a longer-lasting memory. Accordingly, memory formation requires the timely controlled action of different neuronal circuits, neurotransmitters, neuromodulators and molecules that were initially identified by classical forward genetic approaches. Compared to adult *Drosophila*, memory formation was only sporadically analyzed at its larval stage. Here we deconstruct the larval mnemonic organization after aversive olfactory conditioning. We show that after odor-high salt conditioning larvae form two parallel memory phases; a short lasting component that depends on cyclic adenosine 3’5’-monophosphate (cAMP) signaling and *synapsin* gene function. In addition, we show for the first time for *Drosophila* larvae an anesthesia resistant component, which relies on *radish* and *bruchpilot* gene function, protein kinase C activity, requires presynaptic output of mushroom body Kenyon cells and dopamine function. Given the numerical simplicity of the larval nervous system this work offers a unique prospect for studying memory formation of defined specifications, at full-brain scope with single-cell, and single-synapse resolution.

## Author Summary

Learning and memory helps organisms to predict and adapt to events in their environment. Gained experience leaves traces of memory in the nervous system. Yet, memory formation in vertebrates and invertebrates is a highly complex and dynamic process that consists of different phases, which depend on various neuronal and molecular mechanisms. To understand which changes occur in a brain when it learns, we applied a reductionist approach. Instead of studying complex cases, we analyzed learning and memory in *Drosophila* larvae that have a simple brain that is genetically and behaviorally accessible and consists of only about 10,000 neurons. *Drosophila* larvae are able to learn to associate an odor with punishing high salt concentrations. It is therefore possible to correlate changes in larval behavior with molecular events in identifiable neurons after classical olfactory conditioning. We show that under these circumstances larvae form two parallel memory phases; a short lasting component (lSTM) that is molecularly conserved throughout the animal kingdom as it depends on the classical cAMP pathway. In parallel they establish a larval anesthesia resistant memory (lARM) that relies on a different molecular signal. lARM has not been described in larvae before.

## Introduction

Experience leaves traces of memory in the nervous system. This assists organisms to predict and adapt to events in their environment. Both invertebrates and vertebrates possess a variety of different learning mechanisms [1, 2]. Associative learning, for instance, enables animals to draw on past experience to predict the occurrence of food, predators or social partners [3]. Several studies in vertebrates and invertebrates have revealed that associative memories consist of distinct phases, which differ in duration and time of expression. Throughout the animal kingdom, a labile, short-term memory can be distinguished from a robust, long-term memory [4–6]. Long-term memory—in contrast to short-term memory—is resistant to anesthetic disruption and depends on consolidation processes including *de novo* protein synthesis [4, 6–9].

Genetic studies in adult *Drosophila* following olfactory classical conditioning using electric shock as a negative reinforcer have identified distinct temporal memory phases—short-term memory (STM), middle-term memory (MTM), long-term memory (LTM) and a so-called anesthesia-resistant memory (ARM) [10, 11]. STM and MTM are both considered to be unconsolidated whereas ARM and LTM are consolidated forms of memory. The main property of STM and MTM is a dependency on the cyclic adenosine 3’5’-monophosphate (cAMP) pathway [12] as exemplified by early studies of *rutabaga* (*rut*) encoded type I Ca^2+^-dependent adenylyl cyclase (AC1) [13, 14] and *dunce* (*dnc*) encoded type 4 cAMP-specific phosphodiesterase (PDE4) [15–17]. Consolidated LTM and ARM are assumed to be represented by separate molecular pathways [18]. In contrast to ARM formation, LTM requires cAMP response element-binding protein (CREB) dependent transcription and *de-novo* protein synthesis [10, 19, 20]. Nevertheless, ARM is resistant to anesthetic agents [21], which cause retrograde amnesia in both invertebrates and vertebrates [6, 8, 21, 22]. Furthermore ARM formation requires the activity of the *radish* gene [23, 24]. Taken together, in adult *Drosophila* classical conditioning following odor-electric shock reinforcement establishes at least four sequential and/or parallel memory phases (but see also [25] for a further subdivision of ARM). However, there is growing evidence that things are unlikely to be as straightforward as originally envisaged. For example, changing parameters of the training regime, such as feeding state, age of flies, timing of the stimuli and the reinforcing stimulus affects distinct aspects of memory formation and in the most extreme case leads to a mechanistically different type of memory being formed [26–29].

Based on the above described, well-established genetic interventions that have functional implications for adult *Drosophila* we have analyzed memory formation at the larval stage. Although *Drosophila* larvae are able to form olfactory and visual memories [30–41], only a few studies have described larval memory formation in more detail. Larval olfactory memory also consists of different phases [32, 36, 40, 42, 43]. However, some of the studies identified only a short-lasting memory [32, 42], while others studies came to the conclusion that the larval memory consists of both, a short-lasting and a long-lasting component [36, 40, 43]. Furthermore, genetic dissection of the larval memory linked memory formation to the cAMP pathway [32, 36, 40, 42, 43]. However, two of these studies have shown in addition, that *rsh*^*1*^ mutants and *turnip* (*tur*) mutants, which are reduced in protein kinase C (PKC) activity, showed an impairment in larval memory [36, 40]. Recapitulating the appearance of sequential and/or parallel memory phases in larvae is rather difficult, since these molecular processes were suggested to be independent of cAMP signaling.

Here we have deconstructed the larval mnemonic organization after odor-high salt conditioning. Therefore we adapted paradigms from adult *Drosophila*, which allowed us to identify different components of larval memory. We applied (i) a cold shock in order to identify an anesthesia resistant form and (ii) blocked protein synthesis in order to distinguish protein synthesis independent from the protein synthesis dependent forms.

We have shown that depending on the training regime *Drosophila* larvae are capable of forming distinct memory phases. Following odor-high salt training we identify three different specifications. We describe for the first time an anesthesia resistant memory in larvae (lARM) that it is not affected by cold shock treatment and is evident for up to four hours after training. The component (we use this term here as we were not able to distinguish between the acquisition, consolidation and retrieval of lARM) relies on *radish* and *bruchpilot* gene function, as well as presynaptic output of mushroom body Kenyon cells (MB KCs) and dopaminergic signaling. Furthermore, it utilizes the PKC pathway in contrast to traditional cAMP signaling. Second, we describe a short lasting component (evident for up to 20 minutes after one cycle training) that depends on traditional cAMP signaling and *synapsin* gene function. Third, we identify a CREB dependent component that requires a spaced training protocol, which is composed of five cycles of conditioning spaced by rest intervals of 15 minutes.

## Results

### *Drosophila* larvae establish an aversive olfactory memory that lasts several hours

**Fig 1:**
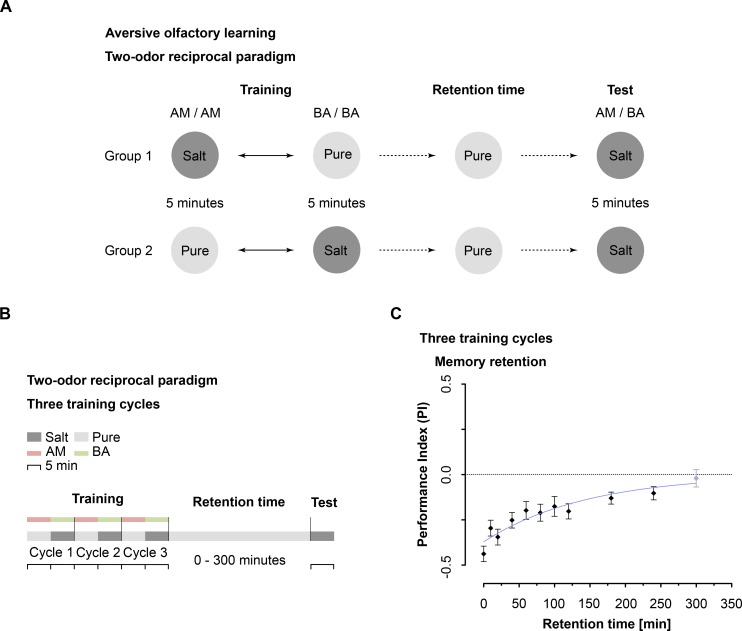
Aversive olfactory memory after odor-high salt conditioning lasts up to four hours **A:** Schematic drawing of the used two odor reciprocal training paradigm. During training, thirty larvae receive the odor n-amylacetate (AM) paired with an aversive reinforcer (high salt concentration) while benzaldehyde (BA) was presented alone (AM^Salt^ / BA^Pure^) (Group 1). Group 2 receives the reverse contingency (AM^Pure^ / BA^Salt^). The training was three times repeated. During test both odors are presented on opposite sides. After 5 minutes the number of larvae on each odor side is counted for both reciprocally trained groups and a performance index (PI) is calculated that quantifies associative olfactory memory. **B:** Flowchart that summarizes the details of the behavioral paradigm in an alternative way. This representation is used throughout the manuscript. Note, for simplification the reciprocally trained group is not shown. **C:** Larval aversive olfactory memory using three training repetitions was tested in wild type larvae at different time points after conditioning ranging from 0–300 minutes. The aversive memory is stable up to four hours (One sample t test, p<0.05 for t = 0-250min; p>0.05 for t = 300min). The memory decay was fitted into an exponential decay function (nonlinear regression analysis, R^2^ = 0.257, τ = -145.9). Memory performance significantly different from random distribution (p<0.05) is indicated in black, random distribution (p≥0.05) in light grey. Sample size is n = 16 for each group. All data are given as means ± s.e.m.

Third instar *Drosophila* larvae are able to learn to associate an odor with punishing high salt concentrations [39, 44]. Thus we utilized a well-established and standardized two odor reciprocal olfactory conditioning paradigm with 1.5M sodium chloride (NaCl) as negative reinforcement and tested memory persistence by assaying larvae at increasing times after training (**Fig 1A**). Please note that the standardized paradigm consists of three training trials (**Fig 1B**). Significant aversive olfactory memory was evident up to four hours after training (**Fig 1C**). However, the memory exhibited a gradual decay as the time interval increased and was no longer statistically significant after five hours (**Fig 1C**). The result is supported by nonlinear regression analysis, which describes the retention curve of odor-high salt memory through an exponential decay function (**Fig 1C**). This suggests that the initially formed odor-high salt memory gradually decays over time.

### Aversive olfactory learning and memory is independent of de-novo protein synthesis and resistant to cold shock

**Fig 2:**
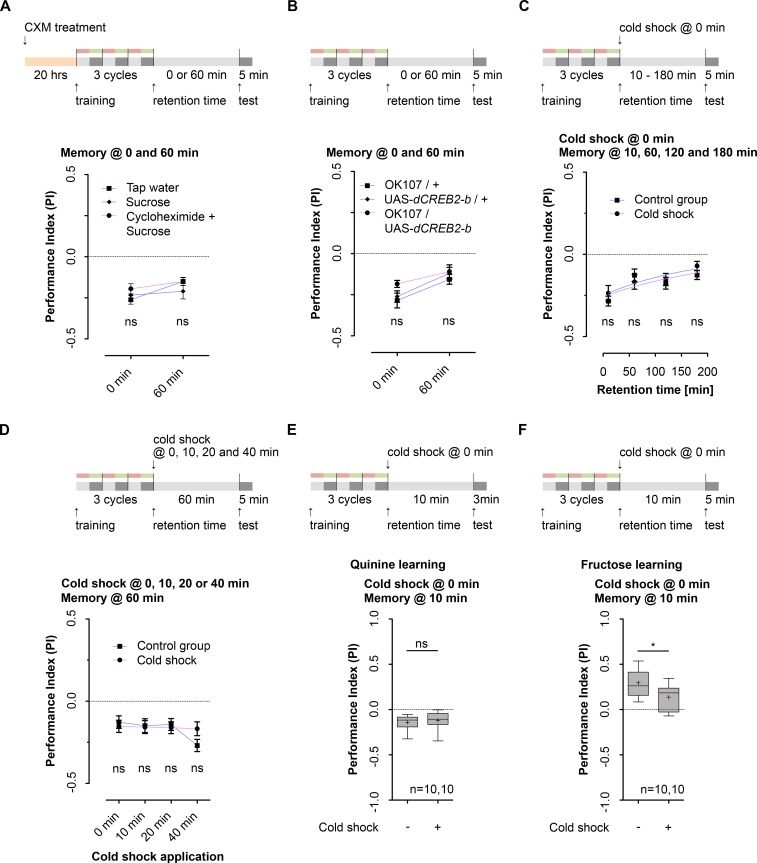
*Drosophila* larvae establish an anesthesia resistant type of memory (lARM) Training and different treatment protocols are shown at the top of each panel. **A:** Effect of cycloheximide (CXM) on larval aversive olfactory memory of wild type larvae tested directly and 60 minutes after three cycle standard training. Application of CXM 20 hours before training did neither affect aversive olfactory memory at 0 minutes nor at 60 minutes after training (Two way ANOVA, p = 0.313). **B:** Expression of a dominant-negative dCREB2-b repressor transgene (*dCREB2-b*) in MB KCs via OK107-Gal4 did not affect larval aversive olfactory memory tested 0 and 60 minutes after three cycle standard training (Two way ANOVA, p = 0.134 between the experimental group OK107-Gal4/UAS-*dCREB2-b* and both control groups). **C:** Effect of cold shock application on the retention of larval aversive olfactory memory. Directly after training wild type larvae received a one minute cold shock at 4°C. The memory was tested 10, 60, 120 and 180 minutes after three cycle standard training. Cold shock application did not reduce aversive olfactory memory at any time point (Two way ANOVA, p = 0.403). **D:** Effect of cold shock treatment on larval aversive olfactory memory tested 60 minutes after three cycle standard training. Cold shock treatment was applied 0, 10, 20 or 40 minutes after conditioning. Cold shock applied at different time points did not reduce aversive olfactory memory of wild type larvae tested 60 minutes after three cycle standard training (Two way ANOVA, p = 0.149). **E:** Cold shock application administered directly after odor-6 mM quinine training did not reduce aversive olfactory memory in wild type larvae tested 10 minutes after three cycle standard training (Unpaired t test, p = 0.610). **F:** Conditioning with 2.0M fructose reward in wild type larvae led to an appetitive olfactory memory, which is partially sensitive to cold shock treatment (Unpaired t test, p = 0.031). Yet, appetitive olfactory memory is not completely vanished (One sample t test, p = 0.026). Sample size is n = 16 for each group if not indicated otherwise. In Fig 2E and 2F differences between groups are depicted above the respective box plots, at which ns indicates p≥0.05 and * p<0.05. Grey boxes indicate a memory performance above chance level (p<0.05). Small circles indicate outliers. In Fig 2A–2D differences between groups are depicted below the symbols, at which ns indicates p≥0.05. Memory performance significantly different from random distribution (p<0.05) is indicated in black. The data in Fig 2A–2D are shown as means ± s.e.m. The data in Fig 2E and 2F are shown as box plots.

Our data show that larvae can associate odors with high salt punishment and that the learning dependent change in behavior lasts several hours. In adult *Drosophila* two types of longer-lasting memories were described, called ARM and LTM. Besides being resistant to anesthetic disruption, ARM is apparently independent of protein synthesis [29]. Yet, LTM formation requires *de novo* protein synthesis [10, 19]. In order to test if the memory is dependent on *de novo* protein synthesis, we fed larvae the translation-inhibitor cycloheximide (CXM) 20 hours before the experiment [10]. Then odor-high salt memory was tested immediately or 60 minutes after three cycle standard training (**Fig 2A**). Performance was unaffected by CXM treatment (**Fig 2A and S1A Fig.**), suggesting that the formed memory is independent of *de novo* protein synthesis. This conclusion is further supported by two additional findings. First, the deleterious effect of blocking protein synthesis using CXM became apparent by constantly feeding CXM over a longer period of time. CXM treated larvae did neither pupate nor eclose in contrast to both control groups (**S1B Fig.**). Second, the transcription factor cAMP response element-binding protein (CREB) is universally required for LTM, and it has been reported that a dominant-negative *dCreb2b* repressor transgene driven by a heat-shock promoter (*hs-dCreb2b*) reduces LTM formation in a heat-shock dependent manner [19, 20]. Expression of *dCreb2b* via OK107-Gal4 specifically in the larval MB Kenyon cells did not change odor-high salt memory tested immediately or 60 minutes after training when compared to both genetic controls **(Fig 2B and S1C Fig)**. Yet, adult *Drosophila* are only capable of forming LTM following a spaced training protocol composed of at least five cycles of conditioning separated by inter-trial intervals of 15 minutes [10, 11, 25]. Therefore we established a spaced training paradigm for larval odor-high salt conditioning (**S1D Fig.**; five training cycles, 15 minutes inter-trial interval). Spaced training induced a learning dependent change of the behavior of two genetic control groups, but not in the behavior of transgenic larvae expressing *dCreb2b* via OK107-Gal4 specifically in the larval MB Kenyon cells (**S1D Fig.**). Thus, the obtained results suggest that the established type of odor-high salt memory is paradigm dependent. However, the prominent component established following three cycle standard training is independent of protein synthesis—and therefore by a general criteria of memory formation not LTM.

Next, we tested whether odor-high salt memory following three cycle standard training is resistant to anesthesia. We established a cold shock treatment protocol. We trained larvae as described before but put them directly into cold water (4°C) for one minute after training. Larvae were then transferred onto a room temperature agar plate to recover and memory was tested after different retention times. As shown in **Fig 2C** (see also **S2C Fig.**) applying a cold shock treatment did not disrupt odor-high salt memory tested 10, 60, 120 and 180 minutes after training (10 minutes is necessary for recovery from the cold). Even applying a stronger cold shock of 5 minutes, which completely paralyzed larvae, did not affect odor-high salt memory (**S2B Fig.**). We also tested whether cold shock treatment applied 0, 10, 20 or 40 minutes after training disrupted 60 minutes memory. Again, no significant defect was revealed (**Fig 2D and S2D Fig**). To test if larval memory following three cycle standard training is in general resistant to cold shock treatment we additionally used 6mM quinine as a negative reinforcer [34, 45] and 2.0M fructose as an appetitive reinforcer (**Fig 2E and 2F**). For both stimuli the established memory was resistant to cold shock treatment. Please note that in case of fructose reinforcement the obtained memory was partially reduced. Implications for larval appetitive olfactory learning and memory are later discussed. All in all, our results show for the first time that larvae independent of the applied reinforcer are able to form a type of anesthesia resistant memory.

### The *radish* gene is necessary for larval anesthesia resistant learning and memory

**Fig 3:**
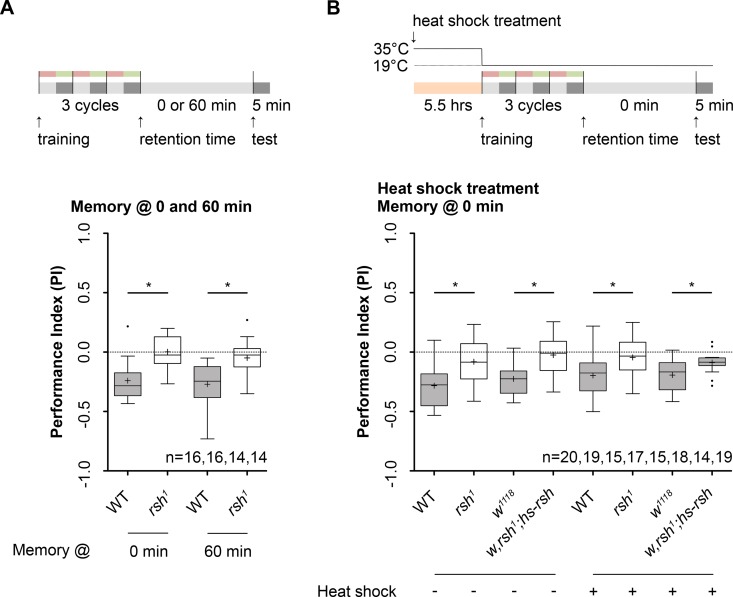
Odor-high salt learning and/or memory depends on *radish* gene function Training and temperature protocols are shown at the top of each panel. **A:** In contrast to wild type larvae, aversive olfactory learning and/or memory is impaired in *rsh*^*1*^ mutants tested 0 and 60 minutes after three cycle standard training (One sample t test, p = 0.95 tested at 0 minutes, p = 0.31 tested at 60 minutes). **B:** Rescue experiment of *rsh*^*1*^ learning and/or memory phenotype using a *hs-rsh* transgene. The transgene was induced via a heat-shock of 35°C for 5.5 hours (continuous line). The control group was kept at 22°C for 5.5 hours (dashed line). Without heat-shock experimental animals (*rsh*^*1*^, *w*,*rsh*^*1*^*;hs-rsh*) tested 0 minutes after three cycle standard training distributed randomly (One sample t test, p = 0.09 for *rsh*^*1*^, p = 0.52 for *w*,*rsh*^*1*^*;hs-rsh*). Yet, both genetic controls showed an aversive memory (One sample t test, p<0.0001 for wild type, Wilcoxon signed rank test, p = 0.0001 for *w*^*1118*^). After heat-shock application only *rsh*^*1*^ mutants distributed randomly (One sample t test, p = 0.25). Yet, ubiquitous induction of *rsh* expression partially rescues the learning and/or memory phenotype (One sample t test, p = 0.0009, unpaired t test, p = 0.01 comparing *w*,*rsh*^*1*^*;hs-rsh* and *w*^*1118*^). Both control groups showed a memory performance above chance level (One sample t test, p<0.0001 for wild type, p<0.0001 for *w*^*1118*^). Differences between groups are depicted above the respective box plots, at which ns indicates p≥0.05 and * p<0.05. Grey boxes show memory performance above chance level (p<0.05), whereas white boxes indicate random distribution (p≥0.05). Small circles indicate outliers. Sample size is n = 16 for each group if not indicated otherwise.

It was shown in adult *Drosophila* that the *radish* (*rsh*) gene plays a pivotal role for the formation of ARM [11, 24]. Hereinafter we therefore focused on *rsh* gene function. We first analyzed the memory performance of *rsh* mutant larvae following three cycle standard training immediately after training or after 60 minutes (**Fig 3A**). In both cases *rsh*^*1*^ mutants showed no significant performance (**Fig 3A)**. To ascertain whether this effect is due to the mutation in the *radish* gene we performed a rescue experiment (**Fig 3B)**. We tested *rsh*^*1*^ mutants that harbor a wild type *rsh* transgene, *hs-rsh*, that allows to induce ubiquitous expression of rsh following heat shock [23]. Non-induced larvae showed a lack of anesthesia resistant learning and/or memory, similar to larvae that carry only the *rsh*^*1*^ mutation. Yet, ubiquitous expression of *rsh* shortly before the experiment rescued the phenotype (**Fig 3B**). However, at a reduced level as compared to wild type controls (**Fig 3B**). Yet, task-relevant sensory-motor abilities of *rsh*^1^ larvae are defective in responding to the odor benzaldeyhde (BA) (**S3B and S3D Fig**). To clearly show that the impairment for *rsh*^*1*^ mutants is based on a loss of the ability to associate odor with high salt concentrations, we performed additional experiments. We used a one odor reciprocal paradigm (**S3C Fig.**) [46]. Here BA presentation is replaced by paraffin oil that does not provide any olfactory information for the larva. Again *rsh*^*1*^ larvae showed no anesthesia resistant learning and/or memory (**S3C Fig.**). In summary, we thus conclude that the behavioral phenotype is due to the fact that the mutation in the *rsh* gene prevents larvae from establishing, consolidating and/or recalling anesthesia resistant memory. Please note that our experiments did not allow to distinguish between the three different processes.

### *Bruchpilot* gene function is necessary at the presynaptic terminals of mushroom body Kenyon cells for anesthesia resistant learning and memory

**Fig 4:**
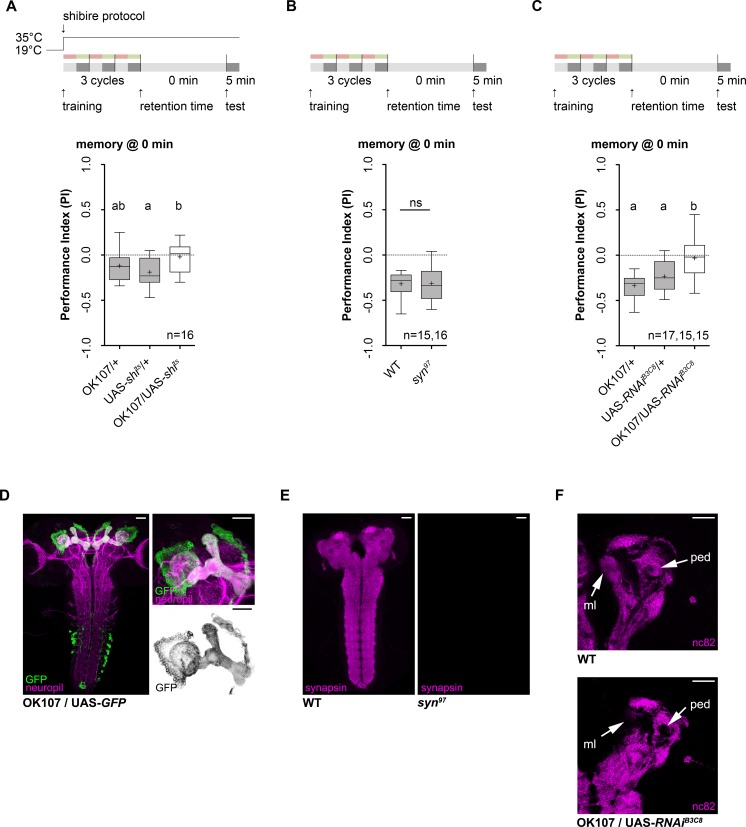
Presynaptic output of mushroom body Kenyon cells is necessary for odor-high salt learning and/or memory Training and temperature shift protocols are shown at the top of each panel. **A:** Effect of the blockade of presynaptic output of MB KCs via UAS-*shi^ts1^* using OK107-Gal4 driver line on odor-high salt learning and/or memory using three cycle standard training. Larvae were raised at the permissive temperature (19°C) and shifted to restrictive temperature during training and testing. In contrast to both genetic controls that show lARM (One sample t test, p = 0.01 for OK107-Gal4/+, p = 0.0003 for UAS-*shi*^*ts1*^/+), *shi*^*ts1*^ expression driven via OK107-Gal4 leads to a complete loss of odor-high salt learning and/or memory tested immediately after three cycle standard training (One sample t test, p = 0.64). Note, only the UAS-*shi*^*ts1*^/+ control but not the OK107-Gal4/+ control was significantly different from the experimental group UAS-*shi*^*ts1*^/OK107-Gal4 (Tukey post hoc test, p = 0.018 and p = 0.22, respectively). **B:** The presynaptic protein Synapsin is not involved in lARM formation. *syn*^*97*^ loss-of-function mutants showed odor-high salt learning and memory (One sample t test, p<0.0001) and behaved non-distinguishable from the genetic control group (Unpaired t test, p = 0.96). **C:** In contrast to both genetic controls (OK107-Gal4/+ and UAS-*brp-RNAi*^*B3C8*^), knockdown of the presynaptic protein *brp* in the KCs by driving UAS-*brp-RNAi*^*B3C8*^ via OK107-Gal4 abolishes odor-high salt learning and/or memory (One sample t test, p = 0.56 for OK107-Gal4/UAS-*brp-RNAi*^*B3C8*^, p<0.0001 for OK107-Gal4/+, p<0.0001 for UAS-*brp-RNAi*^*B3C8*^). **D:** Shows a frontal view projection (left) of a OK107-Gal4/UAS-*mCD8*::*GFP* larval brain labeling the entire set of MB KCs (anti-GFP in green and anti-FasII, anti-ChAT neuropil staining in magenta). Additional staining is detectable in the ventral nerve cord (vnc) and neurons that express *Drosophila* insulin-like proteins (dilp). On the upper right panel a zoom in of the MB is shown. On the lower right panel only the GFP channel is depicted. Scale bars: left panels 50μm, right panels 25μm. **E:** Shows frontal view projections of a wild type brain (left panel) or the *syn*^*97*^ loss-of-function mutant (right panel) stained with anti-synapsin (magenta). As reported, anti-synapsin was only detected in the wild type brain but completely absent in the *syn*^*97*^ loss-of-function mutant. Scale bars: 50μm. **F:** Shows a single section of a frontal view of brain hemispheres of a wild type (left panel) and an experimental larva (right panel) using anti-nc82 to recognize Brp. In contrast to wild type larvae, no anti-nc82 staining was detectable in the MB (shown at the peduncle and the medial lobe level by arrows). Scale bars: 25μm. Differences between groups are depicted above the respective box plots, ns indicates p≥0.05. Different lowercase letters indicate statistical significant differences from each other at level p<0.05. Grey boxes show memory performance above chance level (p<0.05), white boxes indicate random distribution (p≥0.05). Small circles indicate outliers. Sample size is n = 16 for each group if not indicated otherwise

Next we analyzed if intrinsic MB KCs are required for anesthesia resistant learning and/or memory following three cycle standard training due to its conserved role in larval and adult olfactory memory formation [32, 42, 47, 48]. Expression of the temperature-sensitive dominant negative form of dynamin *shibire*^*ts1*^ (UAS-*shi*^*ts1*^) [48, 49] via the OK107-Gal4 in all KCs allows to block synaptic KC output at a restrictive temperature of 35°C due to impaired vesicle recycling (**Fig 4A)**. In contrast to both genetic control groups, OK107-Gal4/UAS-*shi*^*ts1*^ larvae showed no anesthesia resistant learning and/or memory (**Fig 4A**). Yet significant difference was only detectable between the UAS-*shi*^*ts1*^/+ control and OK107-Gal4 /UAS-*shi*^*ts1*^ (**Fig 4A**). Control experiments revealed no gross defects in task-relevant sensory-motor abilities (**S4A Fig.)**. In addition UAS-*mCD8*::*GFP* expression driven by OK107-Gal4 verified MB specificity in all KCs besides a limited expression in the ventral nerve cord and brain hemispheres (**Fig 4D**) [48]. Repetition of the experiment with a second mushroom body specific driver H24-Gal4 [48] verified the results obtained for OK107-Gal4 (**S4C and S4D Fig**). Thus, we conclude that KC output is necessary for anesthesia resistant learning and/or memory.

In adult *Drosophila* two presynaptic determinants, Synapsin (Syn) and Bruchpilot (Brp), play a pivotal role in controlling the release of KC vesicles. The evolutionary conserved phosphoprotein Syn is responsible for building a reserve pool of vesicles necessary to maintain vesicle release under high action potential frequencies [50–53]. Adult *syn*^*97*^ mutants showed a defect in aversive olfactory memory that is independent of ARM formation [54, 55]. The active zone protein Brp, which is a homolog to the ELKS/CAST protein family, is an essential component of the presynaptic dense bodies regulating the release probability of synaptic vesicles [56–58]. The presence of Brp in presynaptic terminals of KCs of adults was suggested to be necessary for establishing ARM [59]. To investigate if both proteins are required for anesthesia resistant learning and/or memory following three cycle standard training, we tested a *syn* deficient mutant *syn*^*97*^ and *brp* specific RNAi knockdown in all MB KCs via OK107-Gal4 (**Fig 4B and 4C**). Gene activity of *syn* was not required for anesthesia resistant learning and/or memory (**Fig 4B**). The performance of *syn*^*97*^ mutants was statistically indistinguishable from wild type larvae that served as a genetic control (**Fig 4B**). Lack of the Syn protein in *syn*^*97*^ was verified using a Syn specific antibody (**Fig 4E**) [47]. In contrast Brp function was necessary for anesthesia resistant learning and/or memory (**Fig 4C**). It was completely absent in OK107-Gal4;UAS-*brp-RNAi*^*B3C8*^ larvae (**Fig 4C**). Cell specific knockdown of *brp* in MB KCs was verified by antibody staining (**Fig 4F**). In addition, b*rp* RNAi knockdown did not reveal gross defects in task-relevant sensory-motor abilities (**S4B Fig.)**. Consequently, we suggest presynaptic activity of the active zone protein Brp in MB KCs is necessary to establish, consolidate and/or retrieve lARM. Please note that our experiments did not allow to distinguish between the three different processes.

### Anesthesia resistant learning and memory is independent of the cAMP/protein kinase A pathway but requires protein kinase C activity

**Fig 5:**
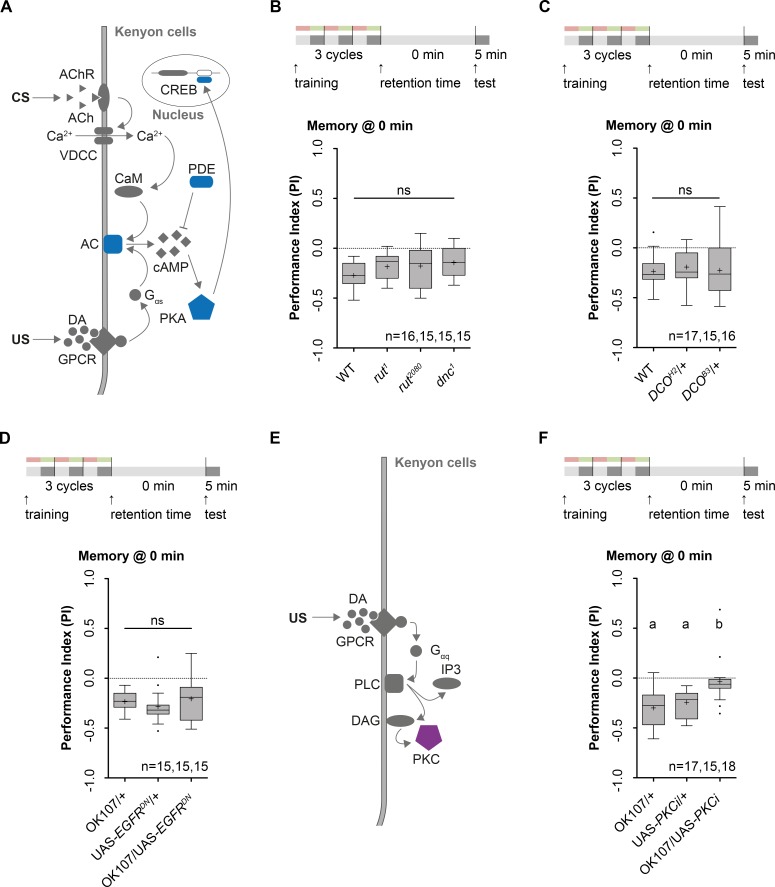
Odor-high salt learning and/or memory following three cycle standard training is independent of the cAMP/PKA pathway Training protocols are shown at the top of each panel. **A:** Working hypothesis that links the classical cAMP/PKA signaling pathway to the association of the unconditioned (US, high salt) and conditioned stimulus (CS, odor). Joined presentation of US and CS activates AC through simultaneous activation of Ca^2+^/CaM and G-protein stimulation. This results in the activation of the cAMP/PKA pathway. The activation of PKA leads either to the phosphorylation of a variety of downstream targets to change neuronal signaling on a shorter time scale or to phosphorylate CREB to form long-lasting memory. An arrowhead indicates stimulatory effects, whereas inhibitory effects are represented as a bar. GPCR: G-protein coupled receptor, VDCC: voltage-dependent calcium channel, ACh: acetylcholine, DA: dopamine, AC: adenylate cyclase, CaM: calcium/calmodulin-dependent protein kinase, cAMP: cyclic AMP, PDE: phosphodiesterase, PKA: protein kinase A, CREB: cyclic AMP response element-binding protein. **B:** *rut*^*1*^, *rut*^*2080*^ and *dnc*^*1*^ mutant larvae showed lARM comparable to wild type controls (One way ANOVA, p = 0.14). **C:** *DCO* encodes for the major catalytic subunit of PKA in *Drosophila*. Adults covering heterozygously the alleles *DCO*^*B3*^/+ and *DCO*^*H2*^/+ show a 50% reduction of PKA activity. *DCO*^*B3*^/+ and *DCO*^*H2*^/+ heterozygous mutant larvae showed normal lARM (One sample t test, p = 0.001 and p = 0.004), they performed at the same level as wild type larvae that served as a genetic control (One way ANOVA, p = 0.84). **D:** Epidermal growth factor receptor (EGFR) signaling to a Ras/Neurofibromatosis type I (NFI) pathway was reported to activate PKA. Expression of a dominant-negative isoform of EGFR (EGFR^DN^) in the MB KCs via OK107-Gal4 does not affect lARM (One sample t test, p = 0.0017), as the experimental group performed at the same level as both genetic controls (Kruskal-Wallis, p = 0.14). **E:** Working hypothesis for an alternative signaling pathway that allows larvae to form lARM. GPCR activation activates PLC that binds to downstream target elements, which stimulate PKC. Activation of typical forms of PKC needs also the binding of intracellular Ca^2+^. An arrowhead indicates stimulatory effects, whereas inhibitory effects are represented as a bar. GPCR: G-protein coupled receptor, DA: dopamine, PLC: phospholipase C, PKC: protein kinase C, DAG: diacylglycerol, IP3: inositoltriphosphat. **F:** Suppression of PKC activity in MB KCs by expressing an inhibitory pseudosubstrate of PKC (PKCi) under the control of OK107-Gal4 leads to a decrease in odor-high salt learning and/or memory (Kruskal-Wallis, p = 0.0001). Differences between groups are depicted above the respective box plots, at which ns indicates p≥0.05. Different lowercase letters indicate statistical significant differences at level p<0.05. Grey boxes show memory performance above chance level (p<0.05), whereas white boxes indicate random distribution (p≥0.05). Small circles indicate outliers. Sample size is n = 16 for each group if not indicated otherwise.

Molecular studies in several model organisms–including *Drosophila*—elucidate cAMP as crucial second messenger in memory formation. A proposed model for the molecular mechanism underlying olfactory memory formation is shown in **Fig 5A**. An association between the odorant and the reinforcement signals elicits an activation of type I Ca^2+^-dependent AC encoded by the *rut* gene via calcium/calmodulin and G-protein stimulation [13, 14, 60]. This synergistic activation of AC produces an increase in intracellular cAMP concentration [12]. Intolerable cAMP concentration is prevented through the activity of a type 4 cAMP-specific PDE encoded by the *dnc* gene [12–14] cAMP for its part activates PKA [61]. The activation of PKA leads either to the phosphorylation of a variety of downstream targets (e.g. Synapsin, Na^+^ and K^+^ channels) for forming a short-lasting memory [47, 62–64] or the phosphorylation of CREB forming a long-lasting memory [19, 20, 65].

To uncover the molecular pathways responsible for anesthesia resistant learning and/or memory following three cycle standard training we tested larvae carrying three classical learning mutations having deficits in cAMP signaling: *rutabaga*^*1*^, *rutabaga*^*2080*^ and *dunce*^*1*^ [14, 17, 24] (**Fig 5B**). All three mutants showed lARM that was indistinguishable from wild type controls (**Fig 5B**). These results indicate that the formation, consolidation and retrieval of lARM is independent of the cAMP/PKA signaling pathway. This conclusion is further supported by two additional findings. First, hypomorphic alleles of the *DCO* gene locus (*DCO*^*B3*^ and DCO^*H2*^*)*, which encodes the major catalytic subunit of the cAMP-dependent PKA (PKAc) showed normal lARM similar to genetic controls (**Fig 5C)**. In adults these heterozygous *DCO*^*B3*^/+ and *DCO*^*H2*^/+ mutants show a 50% reduction of PKA activity and suppress age-related memory impairment [27, 66]. Second, epidermal growth factor receptor (EGFR) signaling to a Ras/Neurofibromatosis type I (NFI) pathway was suggested to act via a Rut-AC independent AC to activate PKA function [67]. Notably pan neuronal expression of a dominant-negative isoform of EGFR (EGRF^DN^) impairs olfactory memory formation of *Drosophila* larvae after bidirectional conditioning [68]. Yet, expression of EGFR^DN^ in all KCs using the OK107-Gal4 did not affect lARM (**Fig 5D**). Please note that our results do not exclude a potential contribution for these genes at later time points after three cycle standard training.

Therefore, biochemical pathways independent of cAMP/PKA signaling cascades have to be involved in lARM tested directly after three cycle standard training. PKC signaling **(Fig 5E)** may serve this function as *tur* mutants that have a reduced PKC activity are impaired in olfactory learning in adult *Drosophila* [69]. Furthermore, expression of a truncated constitutively active isoform of PKC (PKCζ) rescues the memory defects of *rsh*^*1*^ mutants [70]. In fact, transgenic larvae expressing a specific peptide inhibitor of PKC (PKCi) in all KCs using OK107-Gal4 showed strongly reduced anesthesia resistant learning and/or memory in contrast to both genetic controls (**Fig 5F**). Control experiments revealed no gross defects in task-relevant sensory-motor abilities (**S5 Fig.)**. Thus, we conclude that the formation, consolidation and retrieval lARM tested directly after training is independent of the cAMP/PKA pathway and may instead require PKC signaling in KCs.

### Dopamine signaling is necessary for lARM formation

**Fig 6:**
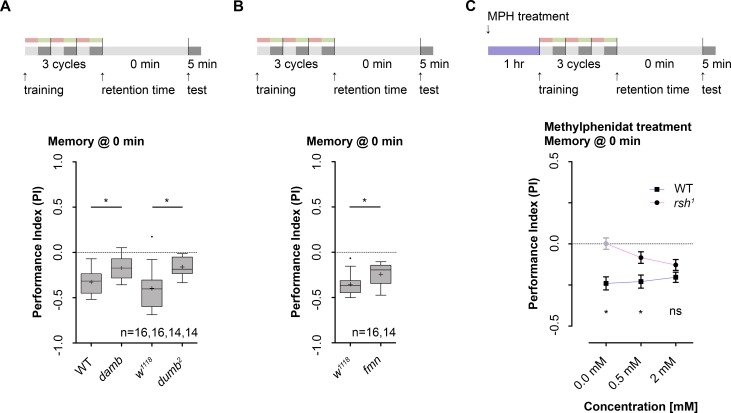
Dopaminergic signaling is necessary for odor-high salt learning and/or memory following three cycle standard training Training and methylphenidate treatment protocols are shown at the top of each panel. **A:** Odor-high salt learning and/or memory was reduced in *damb* as well as in *dumb*^*2*^ dopamine (DA) receptor mutants compared to their respective controls (Unpaired t test, p = 0.002 for *damb*, Mann-Whitney test, p = 0.001 for *dumb*^*2*^). **B:** An increase in DA signaling through mutating the dopamine transporter (DAT) in *fumin* (*fmn*) mutant larvae leads to reduction of odor-high salt learning and/or memory compared to control larvae (Unpaired t test, p = 0.015). **C:** Effect of methylphenidate (MPH) treatment prior of three cycle standard training on odor-high salt learning and/or memory. Larvae were fed MPH for one hour in order to impair DAT function. MPH application was done at a concentration of 0.0 (control), 0.5 and 2.0mM. An acute increase in dopaminergic signaling through a reversible blockage of DAT via MPH application leads to a restoration of odor-high salt learning and/or memory in *rsh*^*1*^ mutants (One sample t test, p = 0.04 for 0.5 mM, p = 0.003 for 2.0 mM). Significant differences between wild type and *rsh*^*1*^ mutants was seen for 0.0 mM and 0.5 mM, but not for 2 mM MPH (Bonferroni post hoc pairwise comparisons, p<0.0001, p = 0.015 and p = 0.412, respectively). Sample size is n = 16 for each group if not indicated otherwise. In Fig **6**A and **6**B differences between groups are depicted above the respective box plots, at which * indicates p<0.05. Grey boxes show memory performance above chance level (p<0.05). Small circles indicate outliers. In Fig **6**C differences between groups are depicted below the symbols, at which ns indicates p≥0.05 and * p<0.05. Memory performance significantly different from random distribution (p<0.05) is indicated in black, random distribution (p≥0.05) in light grey. The data are shown as means ± s.e.m.

The current model for associative learning in *Drosophila* states that during training the unconditioned punishing stimulus is mediated by a specific set of dopaminergic neurons onto MB KCs via G-protein receptor signaling [42, 71–74]. In *Drosophila* the dopamine D1-like receptor family that includes two different dopamine receptors, called dDA1 and DAMB, was reported to be necessary for larval and adult learning [75, 76]. Generally, activation of D1-like receptors was shown to be linked with cAMP/PKA-signaling via Gα_s_ signaling **(Fig 5A)** [77]. Yet more recently it was reported that D1-like receptors also activate phospholipase C (PLC) via the Gα_o_ signaling, which leads to an activation of PKC **(Fig 5E)** [77]. Thus we were wondering if lARM formation depends on dopaminergic signaling. In line with prior results, we found that mutants for both receptor genes *dumb*^*2*^ (for dDA1) and *damb* (for DAMB) show a defect in anesthesia resistant learning and/or memory following three cycles standard training **(Fig 6A, S6A Fig.)** [72]. In addition, *fumin (fmn)* mutant larvae that have a mutation in the dopamine transporter (dDAT) gene [78]—and thus have enhanced DA levels in adults [79]–also show an impairment in anesthesia resistant learning and/or memory **(Fig 6B)** (for further details see **S6B and S6C Fig**). Finally, acute oral administration of methylphenidate (“Ritalin”, MPH) rescued the behavioral phenotype of *rsh*^*1*^ mutant larvae in a dose-dependent manner (**Fig 6C and S6D Fig)**. MPH application in *Drosophila*, similar to its function in humans, increases DA levels by inhibiting the dopamine transporter (dDAT) that mediates dopamine reuptake from the synaptic cleft [80]. In adults it was shown that oral administration of MPH rescues deficits in optomotor responses of *rsh*^*1*^ mutants [81]. Summarizing, three different experiments suggest that dopaminergic signaling is necessary to establish, consolidate and/or retrieve lARM. Please note that–although the effect of DA is likely specific for the establishment of the memory—our experiments did not allow to distinguish between the three different processes.

### One cycle odor-high salt conditioning establishes two distinct memory phases in *Drosophila* larvae

**Fig 7:**
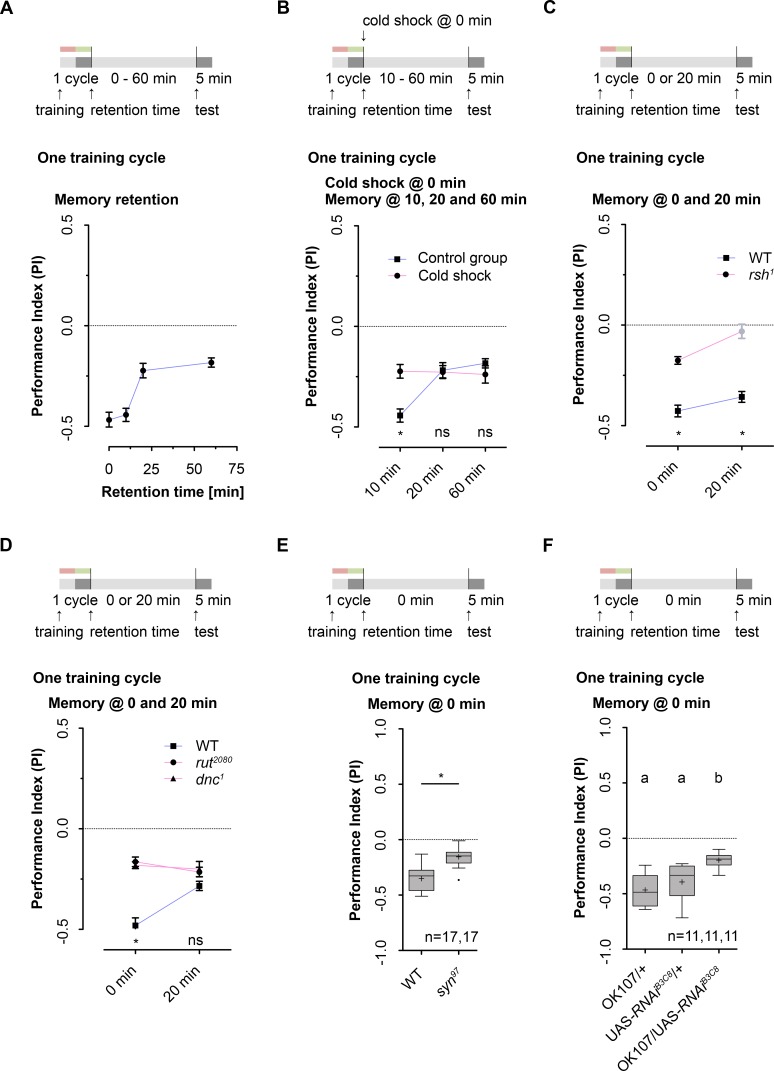
Aversive olfactory memory after one cycle odor-high salt conditioning consist of two different components. Training and cold shock treatment protocols are shown at the top of each panel. **A:** Aversive olfactory memory following one cycle training was tested at different time points after conditioning. 60 minutes after training the memory was still detectable (One sample t test, p<0.0001 for each group). **B:** Cold shock application immediately after one cycle odor-high salt training reduced aversive olfactory learning and/or memory. Yet, the effect was only detectable in comparison to control animals when tested 10 minutes after one cycle training (Bonferroni post hoc pairwise comparisons p<0.05). No difference was seen between both groups 20 and 60 minutes after one cycle training (Bonferroni post hoc pairwise comparisons p>0.05). **C:** *rsh*^*1*^ mutants compared to wild type control animals showed reduced or completely impaired aversive olfactory learning and/or memory after one cycle training (p<0.0001, for both). When tested directly after one cycle training *rsh*^*1*^ mutants showed aversive olfactory memory, however, at a lower level than wild type larvae (Unpaired t test, p<0.0001). **D:** *rut*^*2080*^ and *dnc*^*1*^ mutants compared to wild type control animals showed reduced aversive olfactory learning and/or memory tested immediately after one cycle training (Bonferroni post hoc pairwise comparisons p<0.0001 for both). No difference was detectable for both mutants when tested 20 minutes after conditioning. Furthermore no differences in the memory performance for the mutants were observable between 0 and 20 minutes (p<0.0001 for each comparison). **E:** *syn*^*97*^ loss-of-function mutants showed reduced aversive olfactory learning and/or memory tested immediately after a single-training cycle (Unpaired t test, p<0.0001). However, the mutation in *syn*^*97*^ did not completely abolish aversive olfactory learning and/or memory (One sample t test, p<0.0001). **F:** Compared to both genetic controls (OK107-Gal4/+ and UAS-*brp-RNAi*^*B3C8*^), knockdown of the presynaptic protein *brp* in MB KCs by driving UAS-*brp-RNAi*^*B3C8*^ via OK107-Gal4 reduced aversive olfactory learning and/or memory tested immediately after one cycle training (Kruskal-Wallis, p = 0.0001). In contrast to three cycle standard training, abolishment of aversive olfactory learning and/or memory tested immediately after one cycle training was only partially (One sample t test, p<0.0001). Sample size is n = 16 for each group if not indicated otherwise. In Fig 7B–7D differences between groups are depicted below the data; ns indicated p≥0.05 and * p<0.05. Memory performance significant different from random distribution (p<0.05) is indicated in black, whereas random distribution (p≥0.05) was shown in light grey. The data are shown as means ± s.e.m. In Fig 7E and 7F differences between groups are depicted above the respective box plots; * indicates p<0.05. Different lowercase letters indicated statistical significant differences from each other at level p<0.05. Grey boxes indicate a memory performance above chance level (p<0.05). Small circles indicate outliers.

Conditioning *Drosophila* larvae via three cycle standard training takes about 45 minutes. Yet, two studies on larval aversive olfactory learning suggest that short lasting memory phases exist that are only detectable up to 20 or 50 minutes after training onset [36, 42]. These results could mean that three cycle standard training–routinely used in our previous experiments–based on its temporal dimension does not allow to identify short lasting memories. Therefore we established a one cycle training paradigm that only takes about 10 minutes for conditioning (**Fig 7**). Significant aversive olfactory memory was evident 0, 10, 20 and 60 minutes after training (**Fig 7A and S7A Fig.**). To our surprise, one cycle training increased aversive memory compared to three cycle training (both groups were tested immediately after training) (**S7B Fig.**).

Next, we tested whether odor-high salt memory following one cycle training is resistant to anesthesia. As shown in **Fig 7B** (see also **S7D Fig.**) applying a cold shock treatment did partially disrupt odor-high salt memory tested 10 minutes after training (10 minutes is necessary for recovery from the cold). In contrast, memory tested 20 or 60 minutes after one cycle training was cold shock resistant (**Fig 7B)**. Based on these results we conclude that—independent of the number of training trials—odor-high salt conditioning leads to the formation of lARM. However, at the same time a second short lasting memory is established that can only be detected for up to 30 minutes after training onset. Therefore the short lasting memory can only be analyzed after one cycle training but not in longer lasting protocols using two or three training cycles (**S7C Fig.)**. This conclusion is further supported by two additional findings. First, genetic interference with *rsh* and *brp* gene function, both involved in the formation, consolidation and/or retrieval of lARM, tested immediately after one cycle training only partially impaired the performance of experimental larvae (**Fig 7C and 7F)**. These results are different than the ones obtained following three cycle standard training (**Fig 3 and Fig 4C)** since they imply the presence of a second, lARM independent, memory phase. Second, *rut*^*2080*^, dnc^*1*^ and, *syn*^*97*^ mutants tested immediately following one cycle training performed on a lower level than genetic controls (**Fig 7D and 7E)**. Again, the results are different compared to the ones obtained following three cycle standard training (**Fig 4B and Fig 5B)** and suggest that the formation, consolidation and/or retrieval of a larval short lasting memory (lSTM) under these circumstance depends on cAMP signaling.

## Discussion

### *Drosophila* larvae are able to establish an anesthesia resistant form of memory

Memory formation and consolidation usually describes a chronological order, parallel existence or completion of distinct short-, intermediate- and/or long-lasting memory phases. For example, in honeybees, in *Aplysia*, and also in mammals two longer-lasting memory phases can be distinguished based on their dependence on *de novo* protein synthesis [82–85]. In adult *Drosophila* classical odor-electric shock conditioning establishes two co-existing and interacting forms of memory—ARM and LTM—that are encoded by separate molecular pathways [18].

**Fig 8:**
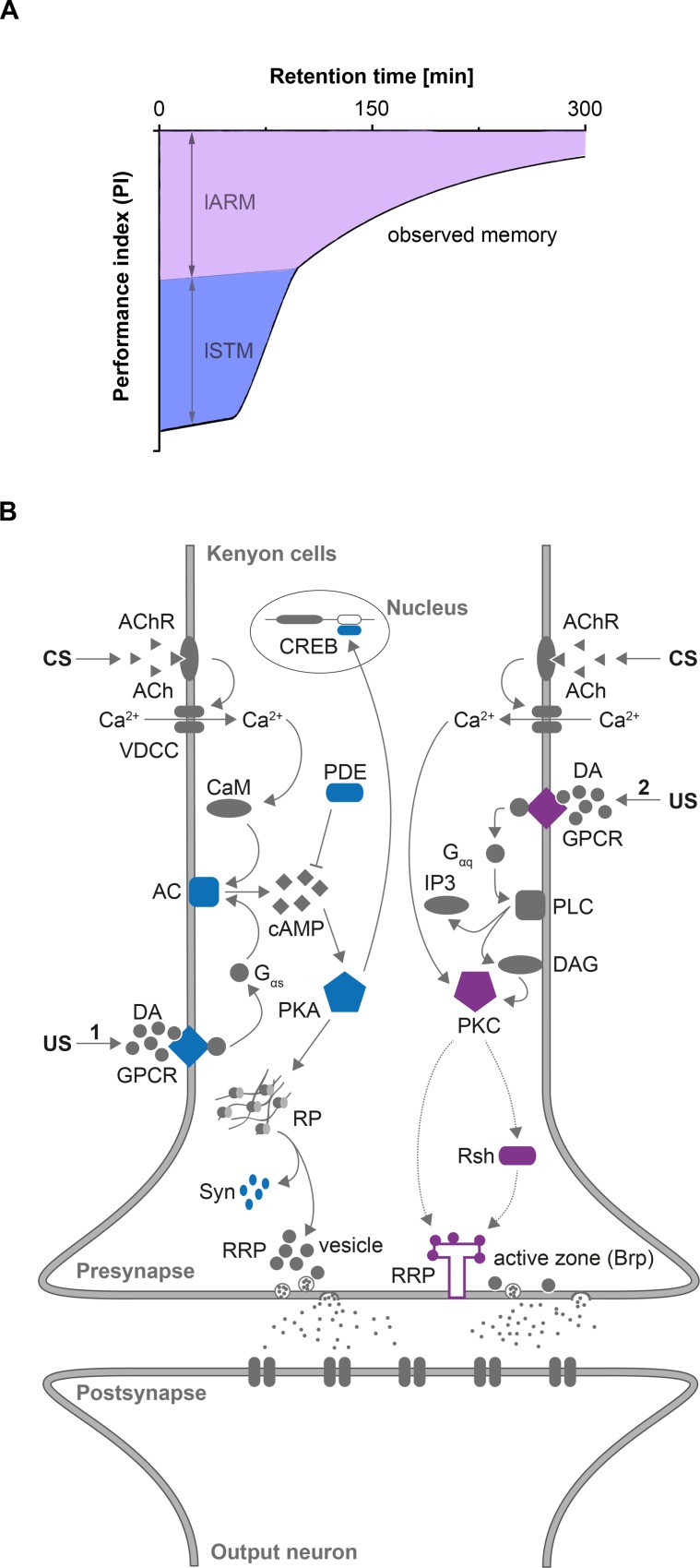
A molecular working hypothesis for lARM formation **A:** Memory formation in *Drosophila* larvae occurs through at least two different components, which are genetically and functionally distinct. First, larval short-term memory (lSTM, light blue) occurs immediately after training, but decays within 20 minutes. Second, larval anesthesia-resistant memory (lARM, light magenta) also appears immediately after training, but lasts for a longer period of time. In contrast to lSTM lARM is resistant to anesthetic disruption. At any given time interval after training the observed memory is the summed output of both components. **B:** Memory formation in *Drosophila* larvae after classical olfactory conditioning depends on protein kinase A (PKA) and protein kinase C (PKC) function, which are involved in two different signaling pathways. During conditioning MB KCs receive an olfactory stimulus via cholinergic projection neurons (conditioned stimulus CS) and a punishing stimulus from dopaminergic neurons (unconditioned stimulus US). In KCs binding of dopamine leads to a dissociation of a G protein subunit from G-protein coupled receptors (GPCR). The CS is perceived by KCs via acetylcholine receptors (AChR). Its activation leads to an opening of a voltage-dependent calcium channel (VDCC) and leads to an intracellular Ca^2+^ influx. This mechanism is thought to be shared between both signaling pathways. In the classical pathway (left side, molecular elements tested in this study are highlighted in light blue), coincident stimulation of CS and US leads to an activation of type I adenylyl cyclase (AC) via Ca^2+^/Calmodulin and dopamine dependent G protein (Gαs) signaling, respectively. Active AC catalyzes the intracellular cAMP production, which is negatively regulated through a phosphodiesterase (PDE) to maintain cAMP concentrations at a tolerable level. cAMP serves as a regulatory signal for PKA, which phosphorylates different substrates like Synapsin (Syn) or CREB in order to induce short or long-lasting presynaptic plasticity. PKC signaling (right side, molecular elements tested in this study are highlighted in light magenta) has been shown in different species as an integral pathway for memory formation. Dopamine receptors were reported to couple to Gαq to regulate phospholipase C (PLC). Activation of PLC increases intracellular inositol triphosphate (IP3) and diacylglycerol (DAG) levels. Whereas IP3 stimulates the release of Ca^2+^ from the endoplasmic reticulum, DAG is a physiological activator of PKC. The downstream elements of PKC are not well described. We suggest that lSTM formation depends on the classical cAMP/PKA pathway, whereas early lARM formation depends on PKC signaling rather than on cAMP/PKA signaling. This assumption is based on the analysis of nine different genes (light purple: lARM phenotype, light blue: lSTM phenotype). In addition, we suggest that PKC can be linked with Radish (Rsh) and Bruchpilot (Brp) as direct or indirect downstream partners. This is so far hypothetical. Yet, a structural analysis on Rsh reported that it has several PKC phosphorylation sites. Ultimately, regulation of Brp via PKC signaling would change the organization of the active zone to provide a molecular substrate for presynaptic plasticity. DA: dopamine, Ach: Acetylcholine, CaM: Calmodulin, RP: reserve pool, RRP: readily releasable pool.

Seen in this light, memory formation in *Drosophila* larvae established via classical odor-high salt conditioning seems to follow a similar logic. It consist of lSTM and lARM **(Fig 8A)** (for a spaced training protocol see also **S1D Fig.**). Aversive olfactory lSTM was already described in two larval studies using different negative reinforcers (electric shock and quinine) and different training protocols (differential and absolute conditioning) [36, 42]. Our results introduce for the first time lARM that was also evident directly after conditioning but lasts longer than lSTM **(Fig 8A)**. lARM was established following different training protocols that varied in the number of applied training cycles **(S7C Fig.)** and the type of negative or appetitive reinforcer **(Fig 2C–2F)**. Thus, lSTM and lARM likely constitute general aspects of memory formation in *Drosophila* larvae that are separated on the molecular level.

### Molecular pathway underlying aversive learning and memory in *Drosophila* larvae

Memory formation depends on the action of distinct molecular pathways that strengthen or weaken synaptic contacts of defined sets of neurons (reviewed in [1, 73, 86–88]). The cAMP/PKA pathway is conserved throughout the animal kingdom and plays a key role in regulating synaptic plasticity. Amongst other examples it was shown to be crucial for sensitization and synaptic facilitation in *Aplysia* [1, 86], associative olfactory learning in adult *Drosophila* and honeybees [85, 88], long-term associative memory and long-term potentiation in mammals [89–92].

For *Drosophila* larvae two studies by Honjo et al. [42] and Khurana et al. [36] suggest that aversive lSTM depends on intact cAMP signaling. In detail, they showed an impaired memory for *rut* and *dnc* mutants following absolute odor-bitter quinine conditioning [42] and following differential odor-electric shock conditioning [36]. Thus, both studies support our interpretation of our results. We argue that odor-high salt training established a cAMP dependent lSTM due to the observed phenotypes of *rut*, *dnc* and *syn* mutant larvae **(Fig 7D and 7E)**. The current molecular model is summarized in **Fig 8B**. Yet, it has to be mentioned that all studies on aversive lSTM in *Drosophila* larvae did not clearly distinguish between the acquisition, consolidation and retrieval of memory. Thus, future work has to relate the observed genetic functions to these specific processes.

In contrast, lARM formation utilizes a different molecular pathway. Based on different experiments, we have ascertained, that lARM formation, consolidation and retrieval is independent of cAMP signaling itself **(Fig 5B)**, PKA function **(Fig 5C),** upstream and downstream targets of PKA **(Figs 5D and 4B)** and *de-novo* protein synthesis **(Fig 2A and 2B)** (but see also for spaced conditioning **S1D Fig.**). Instead we find that lARM formation, consolidation and/or retrieval depends on *rsh* gene function **(Fig 3)**, *brp* gene function **(Fig 4C)**, dopaminergic signaling **(Fig 6)** and requires presynaptic signaling of MB KCs **(Fig 4A and 4C and S4C–S4E Fig.)**.

Interestingly, studies on adult *Drosophila* show that *rsh* and *brp* gene function, as well as dopaminergic signaling and presynaptic MB KC output are also necessary for adult ARM formation [23–25, 59]. Thus, although a direct comparison of larval and adult ARM is somehow limited due to several variables (differences in CS, US, training protocols, test intervals, developmental stages, and coexisting memories), both forms share some genetic aspects. This is remarkable as adult ARM and lARM use different neuronal substrates. The larval MB is completely reconstructed during metamorphosis and the initial formation of adult ARM requires a set of MB α/β KCs that is born after larval life during puparium formation [25, 93, 94].

In addition, we have elicited the necessity of PKC signaling for lARM formation in MB KCs **(Fig 5C)**. The involvement of the PKC pathway for memory formation is also conserved throughout the animal kingdom. For example, it has been shown that PKC signaling is an integral component in memory formation in *Aplysia* [95–98], long-term potentiation and contextual fear conditioning in mammals [99–101] and associative learning in honeybees [102]. In *Drosophila* it was shown that PKC induced phosphorylation cascade is involved in LTM as well as in ARM formation [103]. Although the exact signaling cascade involved in ARM formation in *Drosophila* still remains unclear, we established a working hypothesis for the underlying genetic pathway forming lARM based on our findings and on prior studies in different model organisms (**Fig 8B**). Thereby we do not take into account findings from Horiuchi et al. [66] and Scheunemann et al. [104] in adult *Drosophila*. These studies show that PKA mutants have increased ARM [66] and that *dnc* sensitive cAMP signaling supports ARM [104]. Thus both studies directly link PKA signaling with ARM formation.

### Working hypothesis for lARM formation

It was shown that KCs act on MB output neurons to trigger a conditioned response after training [105, 106]. Work from different insects suggests that the presynaptic output of an odor activated KCs is strengthened if it receives at the same time a dopaminergic, punishment representing signal. Our results support these models as they show that lARM formation requires accurate dopaminergic signaling **(Fig 6)** and presynaptic output of MB KCs **(Fig 4A and 4C)**. Yet, for lARM formation dopamine receptor function seems to be linked with PKC pathway activation **(Fig 5)**. Indeed, in honeybees, adult *Drosophila* and vertebrates it was shown that dopamine receptors can be coupled to Gαq proteins and activate the PKC pathway via PLC and IP3/DAG signaling [107, 108]. As potential downstream targets of PKC we suggest *radish* and *bruchpilot*. Interference with the function of both genes impairs lARM **(Figs 3 and 4C)**. The *radish* gene encodes a functionally unknown protein that has many potential phosphorylation sites for PKA and PKC [23]. Thus considerable intersection between the proteins Rsh and PKC signaling pathway can be forecasted. Whether this is also the case for the *bruchpilot* gene that encodes for a member of the active zone complex remains unknown. The detailed analysis of the molecular interactions has to be a focus of future approaches. Therefore, our working hypothesis can be used to define educated guesses. For instance, it is not clear how the coincidence of the odor stimulus and the punishing stimulus are encoded molecularly. The same is true for ARM formation in adult *Drosophila*. Based on our working hypothesis we can speculate that PKC may directly serve as a coincidence detector via a US dependent DAG signal and CS dependent Ca^2+^ activation.

### Appetitive learning and memory in *Drosophila* larva

Do our findings in general apply to learning and memory in *Drosophila* larvae? To this the most comprehensive set of data can be found on sugar reward learning. *Drosophila* larva are able to form positive associations between an odor and a number of sugars that differ in their nutritional value [31, 32, 47, 109–111]. Using high concentrations of fructose as a reinforcer in a three cycle differential training paradigm (comparable to the one we used for high salt learning and fructose learning **(Figs 1 and 2F)**) Michels et al. [47, 111] found that learning and/or memory in *syn*^*97*^ mutant larvae is reduced to ∼50% of wild type levels. Thus, half of the memory seen directly after conditioning seems to depend on the cAMP-PKA-synapsin pathway. Our results in turn suggest that the residual memory seen in *syn*^*97*^ mutant larvae is likely lARM **(Fig 2F)**. Thus, aversive and appetitive olfactory learning and memory share general molecular aspects. Yet, the precise ratio of the cAMP-dependent and independent components rely on the specificities of the used odor-reinforcer pairings. Two additional findings support this conclusion. First, Kleber et al. [112] recently showed that memory scores in *syn*^*97*^ mutant larvae are only lower than in wild type animals when more salient, higher concentrations of odor or fructose reward are used. Usage of low odor or sugar concentrations does not give rise to a cAMP-PKA-synapsin dependent learning and memory phenotype. Second, Honjo et al. [32] showed that learning and/or memory following absolute one cycle conditioning using sucrose sugar reward is completely impaired in *rut*^*1*^, *rut*^*2080*^ and *dnc*^*1*^ mutants. Thus, for this particular odor-reinforcer pairing only the cAMP pathway seems to be important. Therefore, a basic understanding of the molecular pathways involved in larval memory formation is emerging. Further studies, however, will be necessary in order to understand how *Drosophila* larvae make use of the different molecular pathways with respect to a specific CS/US pairing.

## Materials and Methods

### Fly stocks and maintenance

Fly strains were reared on standard *Drosophila* medium at 25°C or 18°C in constant darkness or with a 14/10 hr light/dark cycle. For behavioral analysis we used *rut*^*1*^, *rut*^*2080*^, *dnc*^*1*^, *rsh*^*1*^ [14, 17, 24] (kindly provided by T. Preat), *DCO*^*B3*^, *DCO*^*H2*^ [27] (kindly provided by M. Saitoe), *fumin* [78] (kindly provided by M. Heisenberg) and *syn*^*97*^ [54, 111] (kindly provided by B. Gerber) mutants. All lines were outcrossed over several generations with wild type CantonS that was used as a genetic control. In addition, we used the two dopamine receptor mutants *damb* and *dumb*^*2*^ and their genetic controls [71]. Note, that in contrast to earlier studies the *damb* mutant was outcrossed to CantonS over several generations. To rescue the *rsh* dependent phenotype by artificial ubiquitous *rsh* expression we used *rsh*^*1*^*; hs-rsh* larvae [23] (kindly provided by T. Zars). To express Gal4 in all larval KCs we used the driver line OK107 [48, 113] (DGRC no.: 106098). UAS-*shi*^*ts*^ was used to acutely block synaptic output from KCs [49] (BDSC No.: 7068). In addition, we used the four effector lines UAS-*EGFR*^*DN*^ [68] (kindly provided by T. Roeder), UAS-*RNAi*^*B3C8*^ [59] (kindly provided by H. Tanimoto), UAS-*PKCi* [114] (kindly provided by B. Brembs) and UAS-*Creb2-b* [115] (kindly provided by S. Waddell).

### Aversive olfactory conditioning

Experiments were conducted on assay plates (85mm diameter, Cat. No.: 82.1472, Sarstedt, Nümbrecht) filled with a thin layer of 2.5% agarose containing either pure agarose (Sigma Aldrich Cat. No.: A5093, CAS No.: 9012-36-6) or agarose plus reinforcer. We used 1.5M and 2.0M sodium chloride (Sigma Aldrich Cat. No.: S7653, CAS No.: 7647-14-5) [44], 2.0M fructose (Sigma Aldrich Cat. No.: 47740, CAS No.: 57-48-7) [110, 116] and 6mM quinine (quinine hemisulfate salt monohydrate, Sigma Aldrich Cat.No.: Q1250, CAS No.: 207671-44-1) [34, 45]. As olfactory stimuli, we used 10 μl amyl acetate (AM, Fluka Cat. No.: 46022; CAS No.: 628-63-7; diluted 1:250 in paraffin oil, Fluka Cat. No.: 76235, CAS No.: 8012-95-1) and benzaldehyde (BA, undiluted; Fluka Cat. No.: 12010, CAS No.: 100-52-7). Odorants were loaded into custom-made Teflon containers (4.5-mm diameter) with perforated lids [109]. Learning ability was tested by exposing a first group of 30 animals to AM while crawling on agarose medium containing in addition sodium chloride as a negative reinforcer. After 5 min, larvae were transferred to a fresh Petri dish in which they were allowed to crawl on pure agarose medium for 5 min while being exposed to BA (AM+/BA). A second group of larvae received the reciprocal training (AM/BA+). If not stated otherwise, three training cycles are used. Depending on the memory retention larvae were transferred onto another agarose plate prior to training and kept for the indicated time before testing the memory. To increase humidity tap water was added. Memory is tested by transferring larvae onto test plates on which AM and BA were presented on opposite sides. For fructose reinforcement the test plates contains pure agarose whereas for sodium chloride and quinine reinforcement agarose plates containing the respective reinforcer are used. After 5 min, individuals were counted as located on the AM side (#AM), the BA side (#BA), or in a 1 cm neutral zone. By subtracting the number of larvae on the BA side from the number of larvae on the AM side, and dividing by the total number of counted individuals (#TOTAL), we determined a preference index for each training group:

(1a) PREF_AM+/BA_ = (# AM—# BA) / # TOTAL
(1b) PREF_AM/BA+_ = (# AM—# BA) / # TOTAL

To measure specifically the effect of associative learning that is of the odor-reinforcement contingency, we then calculated the associative performance index (PI) as the difference in preference between the reciprocally trained larvae:

(2) PI = (PREF_AM+/BA_−PREF_AM/BA+_) / 2

Negative PIs thus represent aversive associative learning, whereas positive PIs indicate appetitive associative learning. Division by 2 ensures scores are bound within (-1; 1).

### Heat-shock treatment

Heat shocks were applied for six hours. Therefore, food vials with six days old larvae were transferred into an incubator at 35°C. We heat-shocked the *w*,*rhs*^*1*^*;hs-rsh* transgenic flies, *rsh*^*1*^ mutants and as controls wild type and *w*^*1118*^ larvae. Afterwards each group received an aversive olfactory training regime at room temperature.

### Cold-shock treatment

For cold-shock experiments larvae were incubated in ice tap water (4°C) for one minute. Larvae were allowed to recover by transferring them onto agarose plates. They started moving within one minute and were kept on the agarose plates at 23°C until testing.

### Cycloheximide treatment

To test if aversive olfactory learning is dependent on *de novo* protein synthesis, wild type larvae were fed 35 mM cycloheximide (+CXM; Sigma Aldrich Cat. No.: C7698; CAS No.: 66-81-9) in 5% sucrose (w/v), 5% sucrose alone (-CXM) or tab water (-CXM, -SUC) for 20 hours before the experiment [10]. Therefore, 300 ml of solution was added into food vials. Before the experiment larvae were gently transferred to an empty Petri dish and washed with tap water before training and testing.

### MPH feeding

Methylphenidate (MPH; Sigma Aldrich Cat. No.: M2892; CAS No.: 298-59-9), was orally administered to the larvae, as it was shown that oral consumption of MPH is sufficient to inhibit the *Drosophila* dopamine transporter [80]. MPH was diluted in tap water with a concentration of 2 mM, stored in the refrigerator and used within 7 days. MPH was applied for one hour to a group of 30 larvae in Petri dishes with an inner diameter of 35 mm (Greiner). Afterwards larvae were gently transferred to an empty Petri dish and washed with tap water before training and testing.

### Acutely blocking synaptic output with *shibire*^*ts*^

To acutely block synaptic output we used UAS-*shi*^*ts1*^ [49]. Immediately before the experiment, larvae were incubated for 2 min in a water-bath at 37°C. The behavioral experiments were then performed as described before, at restrictive temperature of about 35°C in a custom made chamber placed within a fume hood. Control experiments were performed with incubation at room temperature and at permissive temperature of about 23°C.

### Statistical methods

All statistical analyses and visualizations were done with GraphPad Prism 5.0. Groups that did not violate the assumption of normal distribution (Shapiro–Wilk test) and homogeneity of variance (Bartlett's test) were analyzed with parametric statistics: unpaired t-test (comparison between two groups) or Oneway ANOVA followed by planned pairwise comparisons between the relevant groups with a Tukey honestly significant difference HSD post hoc test (comparisons between groups larger than two). Experiments with data that were significantly different from the assumptions above were analyzed with non-parametric tests, such as Mann–Whitney test (comparison between two groups) or Kruskal–Wallis test followed by Dunn's multiple pairwise comparison (comparisons between groups larger than two). To compare single genotypes against chance level, we used One sample t test or Wilcoxon signed rank test. For statistical test concerned with factors equal two or more, two way ANOVA was applied followed by the planned pairwise multiple comparisons (Bonferroni). The significance level of statistical tests was set to 0.05. Figure alignments were done with Adobe Photoshop. Data were presented as box plots, 50% of the values of a given genotype being located within the boxes, whiskers represent the entire set of data. Outliers are indicated as open circles. The median performance index was either indicated as a bold line and the mean as a cross within the box plot or symbol expressed as means ± s.e.m. Unless stated otherwise, all olfactory conditioning experiments are n = 16.

### Immunostaining

Third instar larvae were put on ice and dissected in phosphate-buffered saline (PBS) [71, 117, 118]. Brains were fixed in 3.6% formaldehyde (Merck, Darmstadt) in PBS for 30 min. After eight times rinsing in PBT (PBS with 3% Triton-X 100, Sigma-Aldrich, St. Louis, MO), brains were blocked with 5% normal goat serum (Vector Laboratories, Burlingame, CA) in PBT for 2 hours and then incubated for two days with primary antibodies at 4°C. Before applying the secondary antibodies for two days at 4°C, brains were washed eight times with PBT. After secondary antibody incubation, brains were washed eight times with PBT and mounted in Vectashield (Vector Laboratories, Burlingame, CA) between two cover slips and stored at 4°C in darkness. Images were taken with a Zeiss LSM 510M confocal microscope with x25 or x40 glycerol objectives. The resulting image stacks were projected and analyzed with Image-J (National Institutes of Health, Bethesda, Maryland, http://imagej.nih.gov/ij) software. Contrast and brightness adjustment as well as rotation and organization of images were performed in Photoshop (Adobe Systems Inc., San Jose, CA).

### Antibodies

To analyze the expression pattern of OK107-Gal4 rabbit anti-GFP antibody (A6455, Molecular Probes; 1:1000) and two different mouse antibodies for staining the cholinergic neuropil (ChAT4B1; DSHB, Iowa City, IA, 1:150) and axonal tracts (1d4 anti-Fasciclin 2; DSHB, Iowa City, IA; 1:50) were applied [71, 117]. A specific antibody for the Synapsin protein was used to verify the mutation *syn*^*97*^ (monoclonal mouse anti-syn, 3C11; DSHB, Iowa City, IA, 1:10) [54]. To analyze if the expression level of Bruchpilot is specifically reduced in the MB KCs by driving UAS-*RNAi*^*B3C8*^ via OK107-Gal4, monoclonal mouse anti-nc82 was used (nc82, DSHB, Iowa City, IA, 1:10) [58]. As secondary antibodies goat anti-rabbit IgG Alexa Fluor 488 (A11008, Molecular Probes, 1:200) and goat anti-mouse IgG Alexa Fluor 647 (A21235, Molecular Probes, 1:200) were used.

## Supporting Information

S1 Fig Aversive olfactory learning and/or memory is independent of de-novo protein synthesis. Training and different treatment protocols are shown at the top of each panel. **A:** Cycloheximide (CXM) treatment applied before training did not reduce aversive olfactory learning and/or memory of wild type larvae. For all three groups aversive olfactory learning and/or memory tested immediately after three cycle standard training was significantly different from random distribution (One sample t test, p<0.0001, respectively) and not significantly different from each other (One way ANOVA, p = 0.33). For all three groups aversive olfactory learning and/or memory tested 60 minutes after three cycle standard training was significantly different from random distribution (One sample t test, p<0.0001, p = 0.0004, and p<0.0001, respectively) and not significantly different from each other (Kruskal-Wallis, p = 0.29). **B:** CXM treatment prevented wild type larvae from pupation (after 5 days) and eclosion (after 10 days) after metamorphosis (red line). Control groups (blue lines) that were raised on standard food or on a sucrose diet showed no effect. Results are shown as means and s.e.m. For each group 10 repetitions were done. A significant number of surviving animals is indicated in black (p<0.05), whereas a non-significant number of pupae or flies is marked in light grey (p≥0.05).**C:** Aversive olfactory learning and/or memory was not affected when interfering with CREB function of mushroom body Kenyon cells (MB KCs) using UAS-*dCREb2-b* and OK107-Gal4. Experimental OK107-Gal4/UAS-*dCREB2b* larvae showed learning and/or memory (One sample t test, p<0.0001, respectively) comparable to two genetic controls (Kruskal-Wallis, p = 0.15) when tested immediately after three cycle standard training. Experimental OK107-Gal4/UAS-*dCREB2b* larvae showed learning and memory tested 60 minutes after three cycle training (One sample t test, p = 0.0004, p = 0.006 and p = 0.002, respectively) comparable to two genetic controls (One way ANOVA, p = 0.64). **D:** We trained larvae using a spaced training protocol consisting of five training cycles with 15 minutes rest intervals in between. Aversive olfactory learning and/or memory is completely abolished when interfering with CREB function of MB KCs using UAS-*dCREb2-b*/OK107-Gal4 larvae (One sample t test, p = 0.180). Both genetic controls showed aversive olfactory learning and memory indistinguishable from each other (Tukey post hoc test, p = 0.916). Sample size is n = 16 for each group if not indicated otherwise. In S1A,S1C,S1D Fig. differences between groups are depicted above the respective box plots; ns indicates p≥0.05. Different lowercase letters indicate statistical significant differences from each other at level p<0.05. Grey boxes show memory performance above chance level (p<0.05), whereas white boxes indicate random distribution (p≥0.05). Small circles indicate outliers in S1B Fig.
(TIF)

S2 Fig *Drosophila* larvae establish an anesthesia resistant type of memory following odor-high salt conditioning. Training and different treatment protocols are shown at the top of each panel. **A:** Wild type larvae were trained using the standard three cycle odor-high salt conditioning paradigm. Three different groups were tested: An experimental group received a cold shock (one minute in a 4°C ice water bath) directly after conditioning. A second group received the same cold shock treatment before the conditioning phase. A third group was not cold shocked. Memory was tested after a short recovering phase of 10 minutes after conditioning. **C**old shock treatment before or after three cycle standard training did not affect learning and memory of the larvae (One sample t test, p<0.0001, p = 0.0002, p<0.0001 and p<0.0001, respectively). All three groups performed on the same level (One way ANOVA, p = 0.68). **B:** In addition, we also tested if a harsh cold shock treatment of 5 minutes at 4°C that completely paralyses the larvae affected the performance of the animals. Also under these conditions experimental larvae showed learning and memory that was resistant to cold shock anesthesia (Two way ANOVA, p = 0.07 comparing duration of the cold shock, p = 0.76 comparing if cold shock treatment was applied or not). **C:** Aversive olfactory learning and memory tested at 10, 60, 120 and 180 minutes after conditioning. Experimental groups received a cold shock directly after three cycle standard training. In all four cases cold shock treated larvae behaved on a comparable level as control groups (Unpaired t test, p = 0.4, p = 0.5, p = 0.68 and p = 0.16, respectively). **D:** Aversive olfactory memory was tested 60 minutes after three cycle standard training; experimental groups received a cold shock 0, 10, 20 or 40 minutes after conditioning. In all four cases cold shock treated larvae behaved as control groups (Unpaired t test, p = 0.5, p = 0.88, p = 0.71 and p = 0.79, respectively). Sample size is n = 16 for each group if not indicated otherwise. In S2A,S2C,S2D Fig. differences between groups are depicted above the respective box plots; ns indicates p≥0.05. Grey boxes indicate a performance above chance level (p<0.05). In S2B Fig. differences between groups are depicted below the symbols; ns indicates p≥0.05. A performance significant different from random distribution (p<0.05) is indicated in black. The data are shown as means ± s.e.m.
(TIF)

S3 Fig Sensory acuity tests for larvae with impaired *radish* gene function. **A:** Schematic representation of chemosensory acuity tests. Olfactory perception is analyzed by putting 30 larvae in the middle of a Petri dish with either an amyl acetate (AM) or a benzaldehyde (BA) containing odor container on one side and an empty container (EC) on the other side. After 5 minutes larvae are counted to calculate an olfactory preference index. For gustatory acuity tests, 30 larvae are put in the middle of a Petri dish that contained pure agarose on one side and agarose plus a high salt concentration on the other side. After 5 minutes larvae were counted to calculate a gustatory preference index. **B:** Naive olfactory and gustatory acuity tests for *rsh*^*1*^ mutant and wild type control larvae. Olfactory preference for AM of *rsh*^*1*^ mutant larvae were not different from the one of wild type controls (Unpaired t test, p = 0.30). *rsh*^*1*^ mutant larvae, however, did not show any significant preference for BA (One sample t test, p = 0.23). Gustatory avoidance for high-salt concentration of *rsh*^*1*^ mutant larvae is not different from the one of wild type controls (Unpaired t test, p = 0.84). **C:** Due to the fact that *rsh1* mutants showed an impaired BA preference, we applied a one odor paradigm. In contrast to three cycle standard training, larvae received only AM and instead of BA paraffin oil (no odor information) during odor-high salt conditioning (one odor paradigm). In line with the results for two odor conditioning *rsh*^*1*^ mutant larvae behaved significantly different compared to wild type control larvae (Unpaired t test, p = 0.0001) and showed no learning and/or memory (One sample t test, p = 0.26). The training protocol is shown at the top of the panel. **D:** Naive olfactory and gustatory acuity tests of experimental and control larvae used to rescue *rsh* gene function. Olfactory preference for AM for *rsh*^*1*^ and *w*,*rsh*^*1*^*;hs-rsh* were comparable to their controls at both temperatures (Mann-Whitney test, p = 0.24, p = 0.82, p = 0.22 and p = 0.10, respectively). However, again *rsh*^*1*^ larvae showed significant differences in their BA preference compared to controls (Unpaired t test, p<0.0001, p = 0.49, p<0.0001 and p = 0.68, respectively). High-salt avoidance of *rsh*^*1*^ and *w*,*rsh*^*1*^*;hs-rsh* larvae was indistinguishable from the behavior of controls (Mann-Whitney test, p = 0.06, p = 0.36, p = 0.11 and p = 0.34, respectively). Sample size is n = 16 for each group if not indicated otherwise. Differences between groups are depicted above the respective box plots; ns indicates p≥0.05 and * p<0.05. Grey boxes indicate a memory performance above chance level (p<0.05), whereas white boxes indicate a memory performance at chance level (p≥0.05). Small circles indicate outliers.
(TIF)

S4 Fig Presynaptic output of mushroom body Kenyon cells is not necessary for naïve behaviors towards olfactory and gustatory stimuli. **A:** Sensory acuity tests when interfering with neuronal output of mushroom body Kenyon cells (MB KCs) using UAS-*shi*^*ts1*^ and OK107-Gal4. Experimental (OK107-Gal4 /UAS-*shi*^*ts1*^) and control larvae (OK107-Gal4 /+ and UAS-*shi*^*ts1*^) showed no difference in their naïve responses to AM, BA and high salt (for AM: One way ANOVA, p = 0.25, for BA: Kruskal-Wallis, p = 0.09 and for high salt: One way ANOVA, p = 0.08). **B:** Sensory acuity tests when knocking down *brp* in the MB KCs via UAS-*brp-RNAi*^*B3C8*^ and OK107-Gal4. Experimental (OK107-Gal4/UAS-*brp-RNAi*^*B3C8*^) and control larvae (OK107-Gal4/+, UAS-*brp-RNAi*^*B3C8*^/+) showed no difference in their naïve responses to AM, BA and high salt (Kruskal-Wallis, p = 0.54, p = 0.27 and p = 0.68, respectively). **C:** Blockade of presynaptic output of MB KCs via UAS-*shi*^*ts1*^ using another driver line H24 completely impaired aversive olfactory learning and/or memory. Larvae were raised at the permissive temperature (19°C) and shifted to restrictive temperature before and during three cycle standard training and testing. In contrast to both genetic controls aversive olfactory learning and/or memory tested immediately after three cycle standard training was completely abolished in H24-Gal4/UAS-*shi*^*ts1*^ larvae (One sample t test, p<0.0001 for both control groups and p = 0.853 for H24-Gal4/UAS-*shi*^*ts1*^). **D:** Sensory acuity tests when interfering with neuronal output of mushroom body Kenyon cells (MB KCs) using UAS-*shi*^*ts1*^ and H24-Gal4. Experimental (H24-Gal4/UAS-*shi*^*ts1*^) and control larvae (H24-Gal4/+, UAS-*shi*^*ts1*^/+) showed no difference in their naïve responses to AM, BA and high salt (One way ANOVA, p = 0.517, p = 0.184 and p = 0.753, respectively). **E:** Shows a frontal view projection (left) of a H24-Gal4/UAS-*mCD8*::*GFP* larval hemispheres labeling the entire set of MB KCs (anti-GFP in green and anti-FasII, anti-ChAT neuropil staining in magenta). The observed staining is nearly specific for the larval MB. Below a zoom in of the MB is shown. Further below only the GFP channel is depicted. Scale bars: upper panel 50μm, middle and lower panel 25μm. Sample size is n = 16 for each group if not indicated otherwise. Differences between groups are depicted above and below the respective box plots; ns indicates p≥0.05. Grey boxes indicate a memory performance above chance level (p<0.05). Small circles indicate outliers.
(TIF)

S5 Fig Sensory acuity tests of larvae with impaired PKC function. **A:** Sensory acuity tests when suppressing PKC activity in the MB KCs using an inhibitory pseudo substrate of PKC (*PKCi*). For more details see S3A Fig. The inhibition of PKC did not alter the perception of AM, BA or high salt. Experimental and control groups were indistinguishable from each other (One way ANOVA, p = 0.99, p = 0.40 and p = 0.50, respectively). Sample size is n = 16 for each group if not indicated otherwise. Differences between groups are depicted above or below the respective box plots, ns indicates p≥0.05. Grey boxes indicate a memory performance above chance level (p<0.05). Small circles indicate outliers.
(TIF)

S6 Fig Sensory acuity test for larvae with impaired dopaminergic signaling. Training and methylphenidate (MPH) treatment protocols are shown at the top of each panel. **A:** Sensory acuity tests of the dopamine (DA) receptor mutants *dumb*^*2*^ and *damb*. Both receptors mutants perceived AM, BA and high salt stimuli comparable to controls (for AM: unpaired t test, p = 0.25 and p = 0.83, for BA: Mann-Whitney test, p = 0.07 and unpaired t test, p = 0.41, for high salt: unpaired t test, p = 0.57 and p = 0.28). **B:** Sensory acuity tests of the DAT mutant *fumin* (*fmn*). *fmn* mutant larvae showed no difference in their naïve responses to AM and high salt (Unpaired t test, p = 0.10 and p = 0.64, respectively). However, the naïve response to BA was impaired (Unpaired t test, p = 0.06). **C:** In line with the results for two odor conditioning, *fmn* mutant larvae using a one odor learning paradigm (to omit BA as a sensory stimulus) behaved significantly different compared to wild type control larvae (paired t test, p = 0.0003). They showed no aversive olfactory learning and/or memory (One sample t test, p = 0.06). **D:** Aversive olfactory memory in *rsh*^*1*^ and wild type control larvae after methylphenidate (MPH) treatment using different concentrations. Memory was tested directly after three cycle standard training. Aversive olfactory learning and memory was indistinguishable from random distribution for the *rsh*^*1*^ mutant without MPH application (One sample t test, p = 0.94). After application of 0.5 mM MPH *rsh*^*1*^ mutant larvae showed reduced learning and/or memory compared to wild type controls (One sample t test, p = 0.03, unpaired t test, p = 0.01). After application of 2.0 mM MPH *rsh*^*1*^ mutant larvae performed as wild type controls (Unpaired t test, p = 0.11). Sample size is n = 16 for each group if not indicated otherwise. Differences between groups are depicted above or below the respective box plots; ns indicates p≥0.05 and * p<0.05. Grey boxes indicate a memory performance above chance level (p<0.05), whereas white boxes indicate a memory performance at chance level (p≥0.05). Small circles indicate outliers.
(TIF)

S7 Fig Aversive olfactory high salt reinforced learning and memory using only one training trial. Training and cold shock treatment protocols are shown at the top of each panel. **A:** Aversive olfactory learning and/or memory using a one cycle training protocol was tested at four different retention times after conditioning (0, 10, 20 and 60 minutes). Statistical significant differences were revealed between the groups (One way ANOVA, p<0.0001). The performance indices measured immediately and 10 minutes after one cycle training were indistinguishable from each other (Tukey post hoc test, p = 0.945). Alike the performance indices measured 20 and 60 minutes after once cycle training showed no statistical significance differences (Tukey post hoc test, p = 0.874). However the performance indices measured 0 and 10 minutes after conditioning where at a higher level than the ones measured 20 and 60 minutes after conditioning when analyzed with Tukey post hoc test (p<0.0001 for 0 and 20, p<0.0001 for 0 and 60, p = 0.0001 for 10 and 20 and p<0.0001 for 10 and 60). **B:** Aversive olfactory learning and memory established with increasing training cycles (One cycle, two cycles and three cycles) revealed significant differences between one cycle training and two or three cycle training (One way ANOVA, p = 0.003; Tukey post hoc test p = 0.037, p = 0.033, respectively). For two and three cycle training no difference was detected (p = 0.705). **C:** Cold shock treatment directly after training partially impaired aversive olfactory learning and/or memory when tested 10 minutes after one cycle training. No difference was detected between cold shock treated and control larvae tested 10 minutes after two and three cycle training (Unpaired t-test, p = 0.0001 for one training cycle, p = 0.623 for two training cycles and p = 0.396 for three training cycles). **D:** Aversive olfactory learning and memory of cold shock treated larvae and control groups tested 10, 20 and 60 minutes after one cycle training. Only cold shocked treated larvae tested 10 minutes after one cycle training showed a significant reduction compared to controls that perceived no cold shock. No effect was seen between cold shocked and control groups tested 20 and 60 minutes after one cycle training (Unpaired t test, p = 0.0001 for 10 minutes, p = 0.934 for 20 minutes and p = 0.681 for 60 minutes). **E:** Aversive olfactory learning and/or memory of *rsh*^*1*^ mutants measured immediately and 20 minutes after one cycle training was reduced compared to wild type larvae (Unpaired t test, p<0.0001 for both). Furthermore, *rsh*^*1*^ mutant revealed a complete loss of aversive olfactory learning and/or memory only when measured 20 minutes after one cycle training but not when measured immediately after one cycle training (One sample t-test, p = 0.383). **F:** Aversive olfactory learning and/or memory of *rut*^*2080*^ and *dnc*^*1*^ mutants was reduced compared to wild type larvae when measured directly after one cycle training (One way ANOVA, p<0.0001; Tukey post hoc test, p<0.0001 for both compared to wild type control and p = 0.902 compared to each other). For both mutants learning and memory was not completely abolished (One sample t test, p<0.0001 for both). Aversive olfactory learning and memory of both mutants tested 20 minutes after one cycle training was indistinguishable from the one of wild type larvae (One way ANOVA, p = 0.106).
(TIF)
