# Supplementary figures and images for: Genetic Dissection of Aversive Associative Olfactory Learning and Memory in Drosophila Larvae
Source: PLoS Genet. 2016 Oct 21;12(10):e1006378. doi: 10.1371/journal.pgen.1006378 (PMC5074598; doi:10.1371/journal.pgen.1006378)

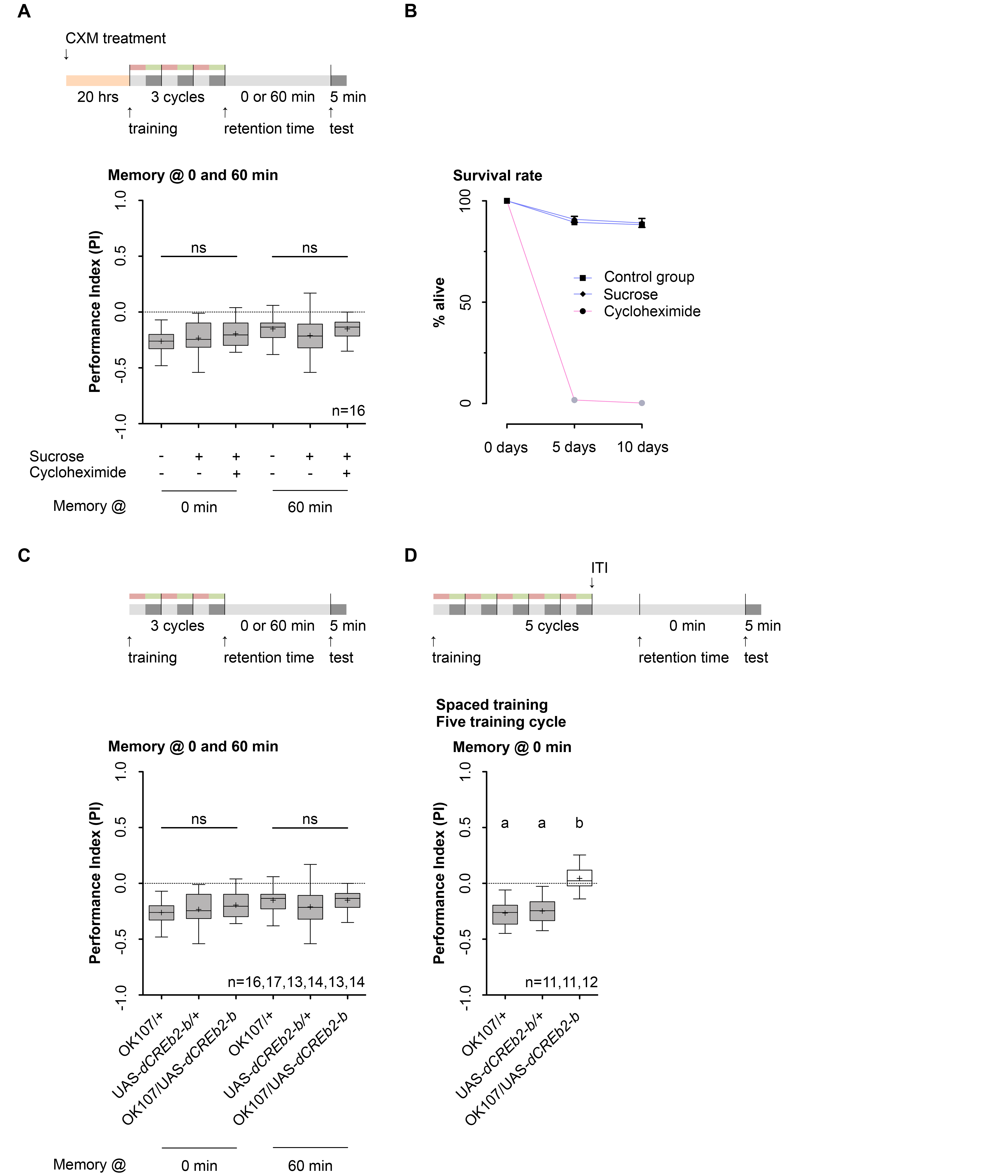

Supplement: S1 Fig — Training and different treatment protocols are shown at the top of each panel. A: Cycloheximide (CXM) treatment applied before training did not reduce aversive olfactory learning and/or memory of wild type larvae. For all three groups aversive olfactory learning and/or memory tested immediately after three cycle standard training was significantly different from random distribution (One sample t test, p<0.0001, respectively) and not significantly different from each other (One way ANOVA, p = 0.33). For all three groups aversive olfactory learning and/or memory tested 60 minutes after three cycle standard training was significantly different from random distribution (One sample t test, p<0.0001, p = 0.0004, and p<0.0001, respectively) and not significantly different from each other (Kruskal-Wallis, p = 0.29). B: CXM treatment prevented wild type larvae from pupation (after 5 days) and eclosion (after 10 days) after metamorphosis (red line). Control groups (blue lines) that were raised on standard food or on a sucrose diet showed no effect. Results are shown as means and s.e.m. For each group 10 repetitions were done. A significant number of surviving animals is indicated in black (p<0.05), whereas a non-significant number of pupae or flies is marked in light grey (p≥0.05).C: Aversive olfactory learning and/or memory was not affected when interfering with CREB function of mushroom body Kenyon cells (MB KCs) using UAS-dCREb2-b and OK107-Gal4. Experimental OK107-Gal4/UAS-dCREB2b larvae showed learning and/or memory (One sample t test, p<0.0001, respectively) comparable to two genetic controls (Kruskal-Wallis, p = 0.15) when tested immediately after three cycle standard training. Experimental OK107-Gal4/UAS-dCREB2b larvae showed learning and memory tested 60 minutes after three cycle training (One sample t test, p = 0.0004, p = 0.006 and p = 0.002, respectively) comparable to two genetic controls (One way ANOVA, p = 0.64). D: We trained larvae using a spaced training pro [file pgen.1006378.s001.tif]

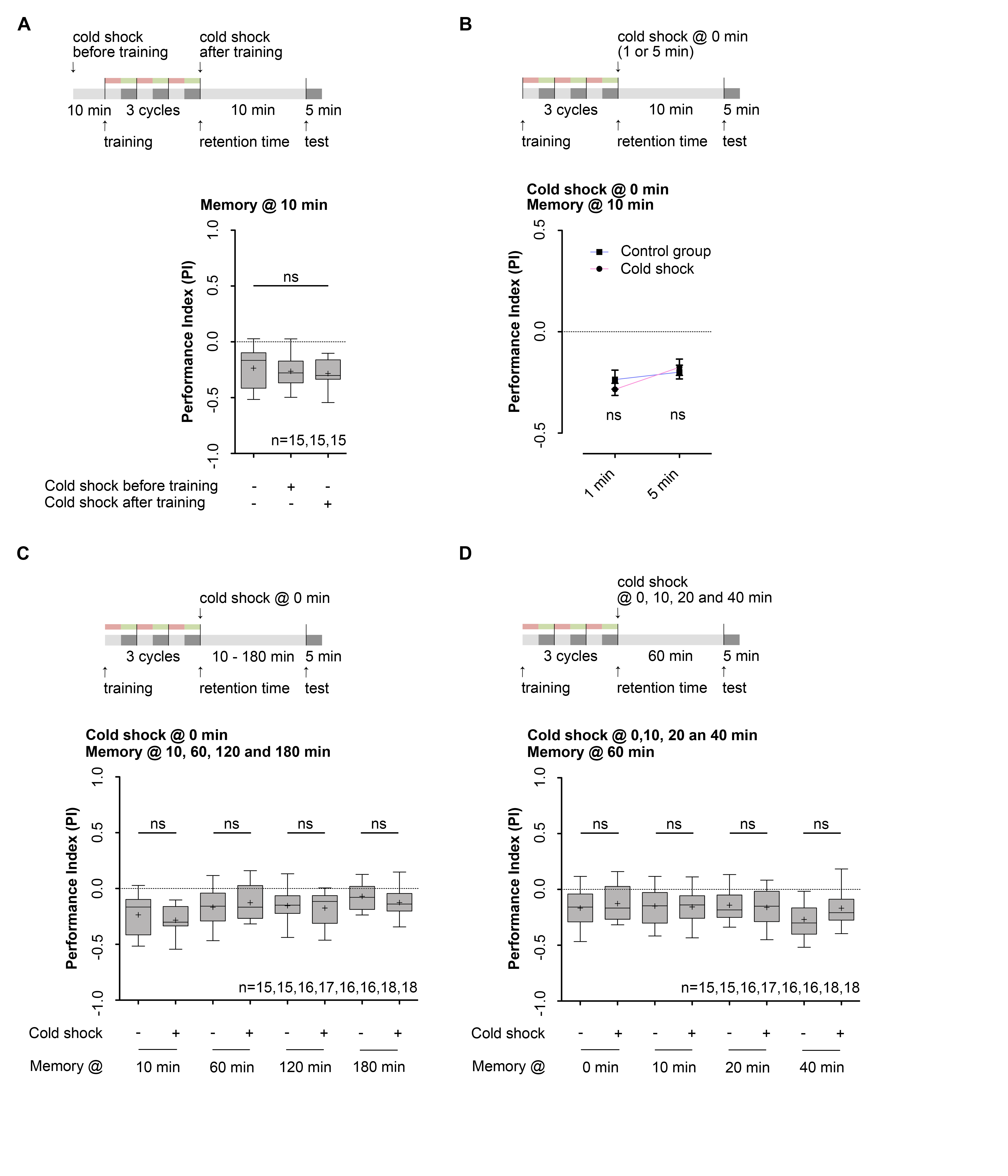

Supplement: S2 Fig — Training and different treatment protocols are shown at the top of each panel. A: Wild type larvae were trained using the standard three cycle odor-high salt conditioning paradigm. Three different groups were tested: An experimental group received a cold shock (one minute in a 4°C ice water bath) directly after conditioning. A second group received the same cold shock treatment before the conditioning phase. A third group was not cold shocked. Memory was tested after a short recovering phase of 10 minutes after conditioning. Cold shock treatment before or after three cycle standard training did not affect learning and memory of the larvae (One sample t test, p<0.0001, p = 0.0002, p<0.0001 and p<0.0001, respectively). All three groups performed on the same level (One way ANOVA, p = 0.68). B: In addition, we also tested if a harsh cold shock treatment of 5 minutes at 4°C that completely paralyses the larvae affected the performance of the animals. Also under these conditions experimental larvae showed learning and memory that was resistant to cold shock anesthesia (Two way ANOVA, p = 0.07 comparing duration of the cold shock, p = 0.76 comparing if cold shock treatment was applied or not). C: Aversive olfactory learning and memory tested at 10, 60, 120 and 180 minutes after conditioning. Experimental groups received a cold shock directly after three cycle standard training. In all four cases cold shock treated larvae behaved on a comparable level as control groups (Unpaired t test, p = 0.4, p = 0.5, p = 0.68 and p = 0.16, respectively). D: Aversive olfactory memory was tested 60 minutes after three cycle standard training; experimental groups received a cold shock 0, 10, 20 or 40 minutes after conditioning. In all four cases cold shock treated larvae behaved as control groups (Unpaired t test, p = 0.5, p = 0.88, p = 0.71 and p = 0.79, respectively). Sample size is n = 16 for each group if not indicated otherwise. In S2A,S2C,S2D Fig. differences between groups are depic [file pgen.1006378.s002.tif]

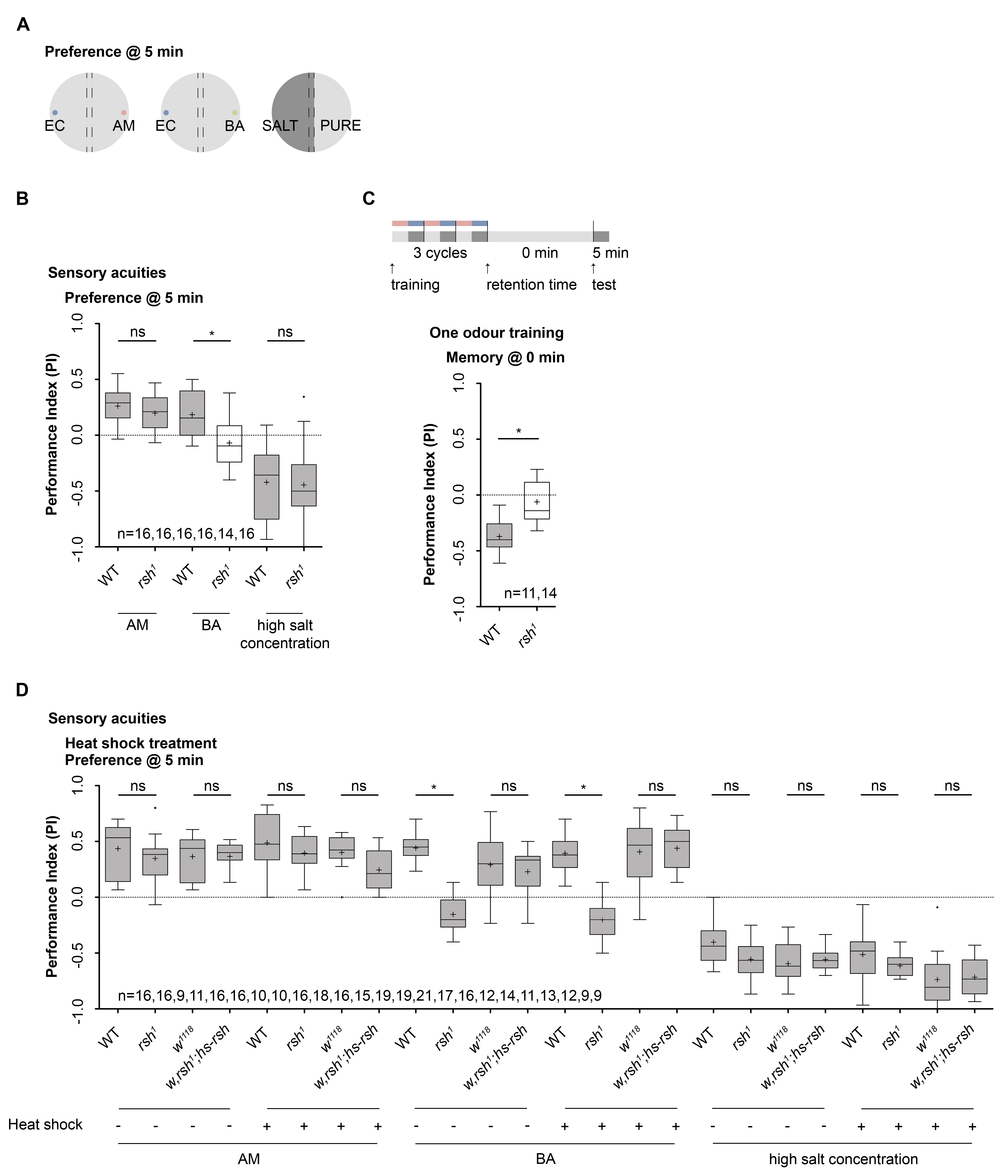

Supplement: S3 Fig — A: Schematic representation of chemosensory acuity tests. Olfactory perception is analyzed by putting 30 larvae in the middle of a Petri dish with either an amyl acetate (AM) or a benzaldehyde (BA) containing odor container on one side and an empty container (EC) on the other side. After 5 minutes larvae are counted to calculate an olfactory preference index. For gustatory acuity tests, 30 larvae are put in the middle of a Petri dish that contained pure agarose on one side and agarose plus a high salt concentration on the other side. After 5 minutes larvae were counted to calculate a gustatory preference index. B: Naive olfactory and gustatory acuity tests for rsh1 mutant and wild type control larvae. Olfactory preference for AM of rsh1 mutant larvae were not different from the one of wild type controls (Unpaired t test, p = 0.30). rsh1 mutant larvae, however, did not show any significant preference for BA (One sample t test, p = 0.23). Gustatory avoidance for high-salt concentration of rsh1 mutant larvae is not different from the one of wild type controls (Unpaired t test, p = 0.84). C: Due to the fact that rsh1 mutants showed an impaired BA preference, we applied a one odor paradigm. In contrast to three cycle standard training, larvae received only AM and instead of BA paraffin oil (no odor information) during odor-high salt conditioning (one odor paradigm). In line with the results for two odor conditioning rsh1 mutant larvae behaved significantly different compared to wild type control larvae (Unpaired t test, p = 0.0001) and showed no learning and/or memory (One sample t test, p = 0.26). The training protocol is shown at the top of the panel. D: Naive olfactory and gustatory acuity tests of experimental and control larvae used to rescue rsh gene function. Olfactory preference for AM for rsh1 and w,rsh1;hs-rsh were comparable to their controls at both temperatures (Mann-Whitney test, p = 0.24, p = 0.82, p = 0.22 and p = 0.10, respectively). However, again rsh1 [file pgen.1006378.s003.tif]

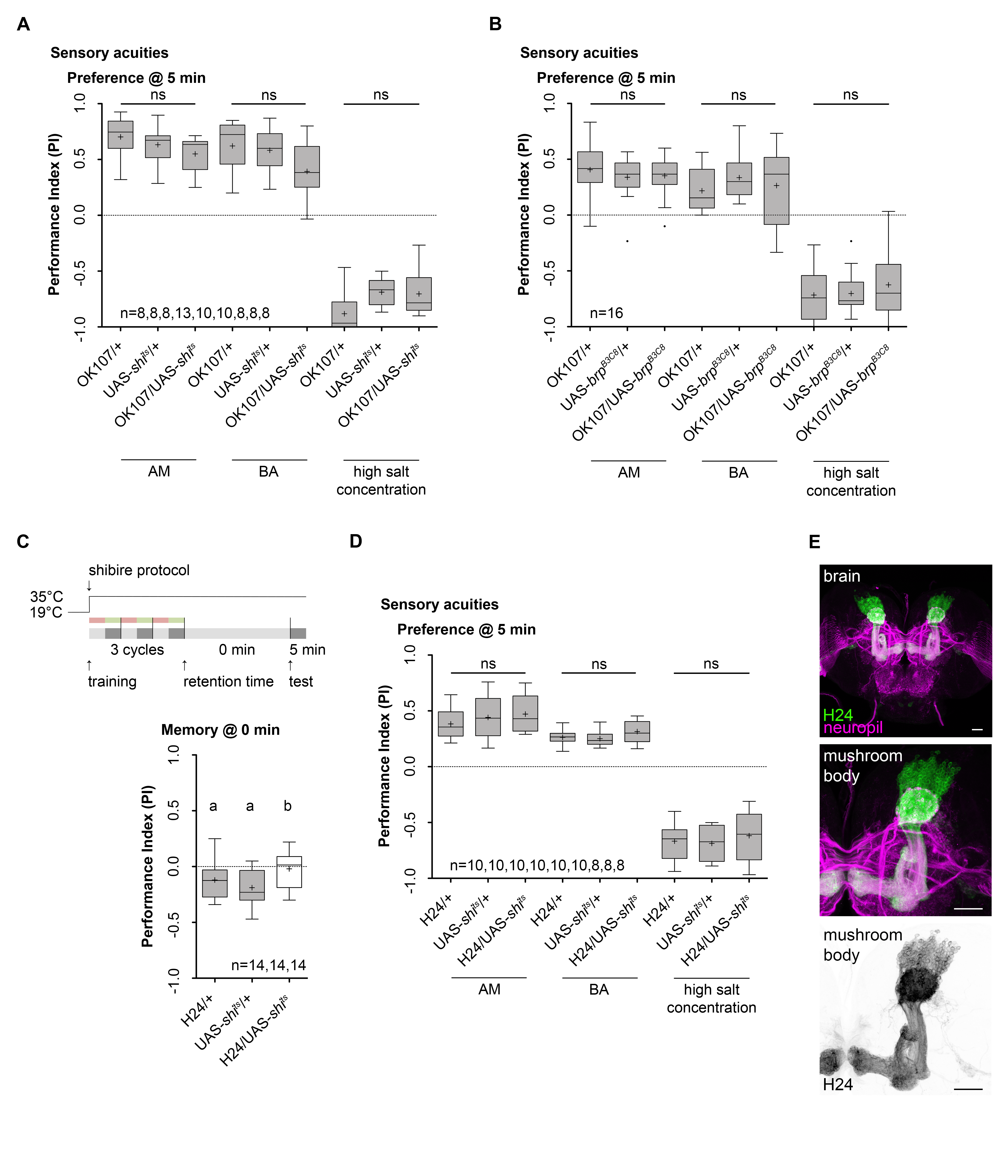

Supplement: S4 Fig — A: Sensory acuity tests when interfering with neuronal output of mushroom body Kenyon cells (MB KCs) using UAS-shits1 and OK107-Gal4. Experimental (OK107-Gal4 /UAS-shits1) and control larvae (OK107-Gal4 /+ and UAS-shits1) showed no difference in their naïve responses to AM, BA and high salt (for AM: One way ANOVA, p = 0.25, for BA: Kruskal-Wallis, p = 0.09 and for high salt: One way ANOVA, p = 0.08). B: Sensory acuity tests when knocking down brp in the MB KCs via UAS-brp-RNAiB3C8 and OK107-Gal4. Experimental (OK107-Gal4/UAS-brp-RNAiB3C8) and control larvae (OK107-Gal4/+, UAS-brp-RNAiB3C8/+) showed no difference in their naïve responses to AM, BA and high salt (Kruskal-Wallis, p = 0.54, p = 0.27 and p = 0.68, respectively). C: Blockade of presynaptic output of MB KCs via UAS-shits1 using another driver line H24 completely impaired aversive olfactory learning and/or memory. Larvae were raised at the permissive temperature (19°C) and shifted to restrictive temperature before and during three cycle standard training and testing. In contrast to both genetic controls aversive olfactory learning and/or memory tested immediately after three cycle standard training was completely abolished in H24-Gal4/UAS-shits1 larvae (One sample t test, p<0.0001 for both control groups and p = 0.853 for H24-Gal4/UAS-shits1). D: Sensory acuity tests when interfering with neuronal output of mushroom body Kenyon cells (MB KCs) using UAS-shits1 and H24-Gal4. Experimental (H24-Gal4/UAS-shits1) and control larvae (H24-Gal4/+, UAS-shits1/+) showed no difference in their naïve responses to AM, BA and high salt (One way ANOVA, p = 0.517, p = 0.184 and p = 0.753, respectively). E: Shows a frontal view projection (left) of a H24-Gal4/UAS-mCD8::GFP larval hemispheres labeling the entire set of MB KCs (anti-GFP in green and anti-FasII, anti-ChAT neuropil staining in magenta). The observed staining is nearly specific for the larval MB. Below a zoom in of the MB is shown. Further below only the GFP chan [file pgen.1006378.s004.tif]

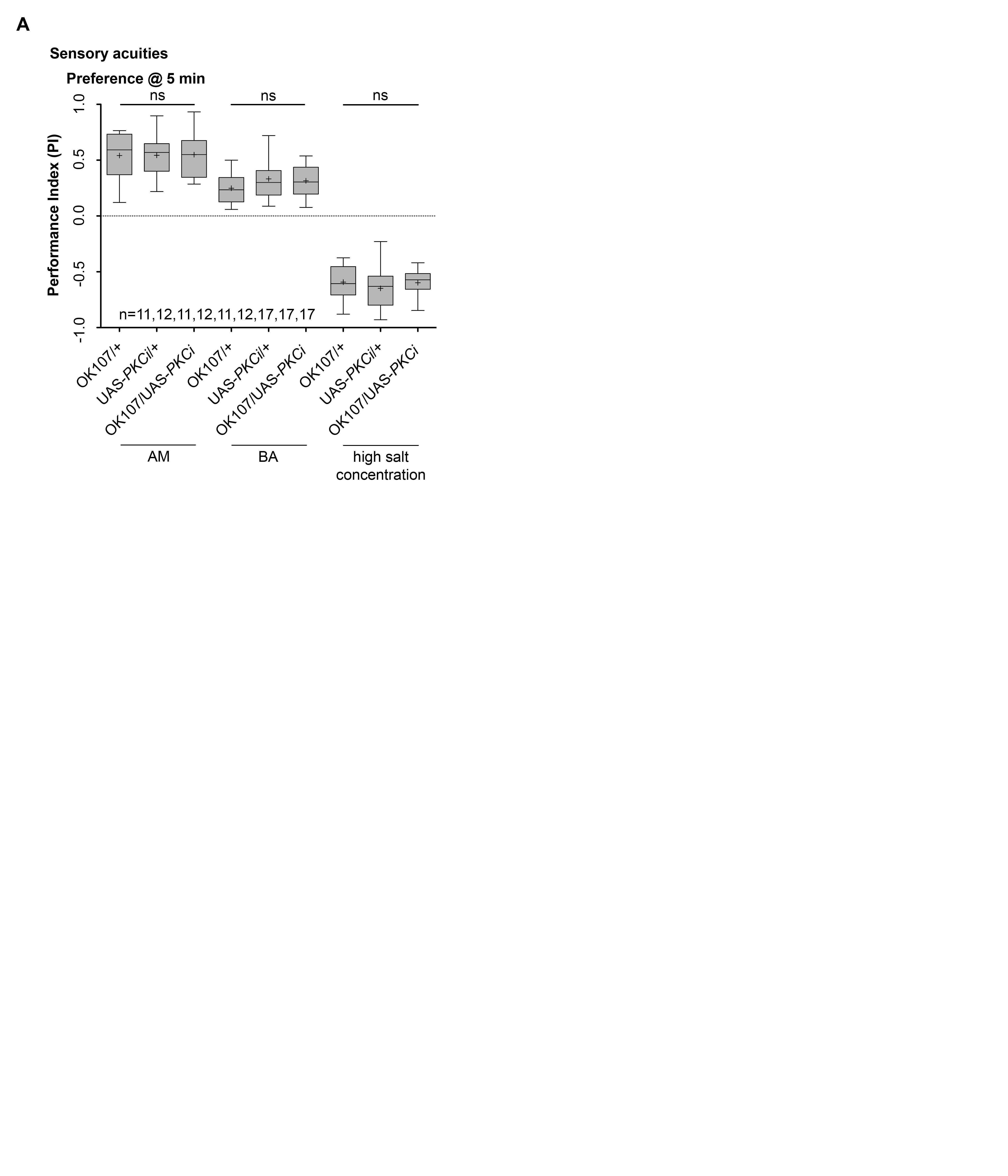

Supplement: S5 Fig — A: Sensory acuity tests when suppressing PKC activity in the MB KCs using an inhibitory pseudo substrate of PKC (PKCi). For more details see S3A Fig. The inhibition of PKC did not alter the perception of AM, BA or high salt. Experimental and control groups were indistinguishable from each other (One way ANOVA, p = 0.99, p = 0.40 and p = 0.50, respectively). Sample size is n = 16 for each group if not indicated otherwise. Differences between groups are depicted above or below the respective box plots, ns indicates p≥0.05. Grey boxes indicate a memory performance above chance level (p<0.05). Small circles indicate outliers. (TIF) [file pgen.1006378.s005.tif]

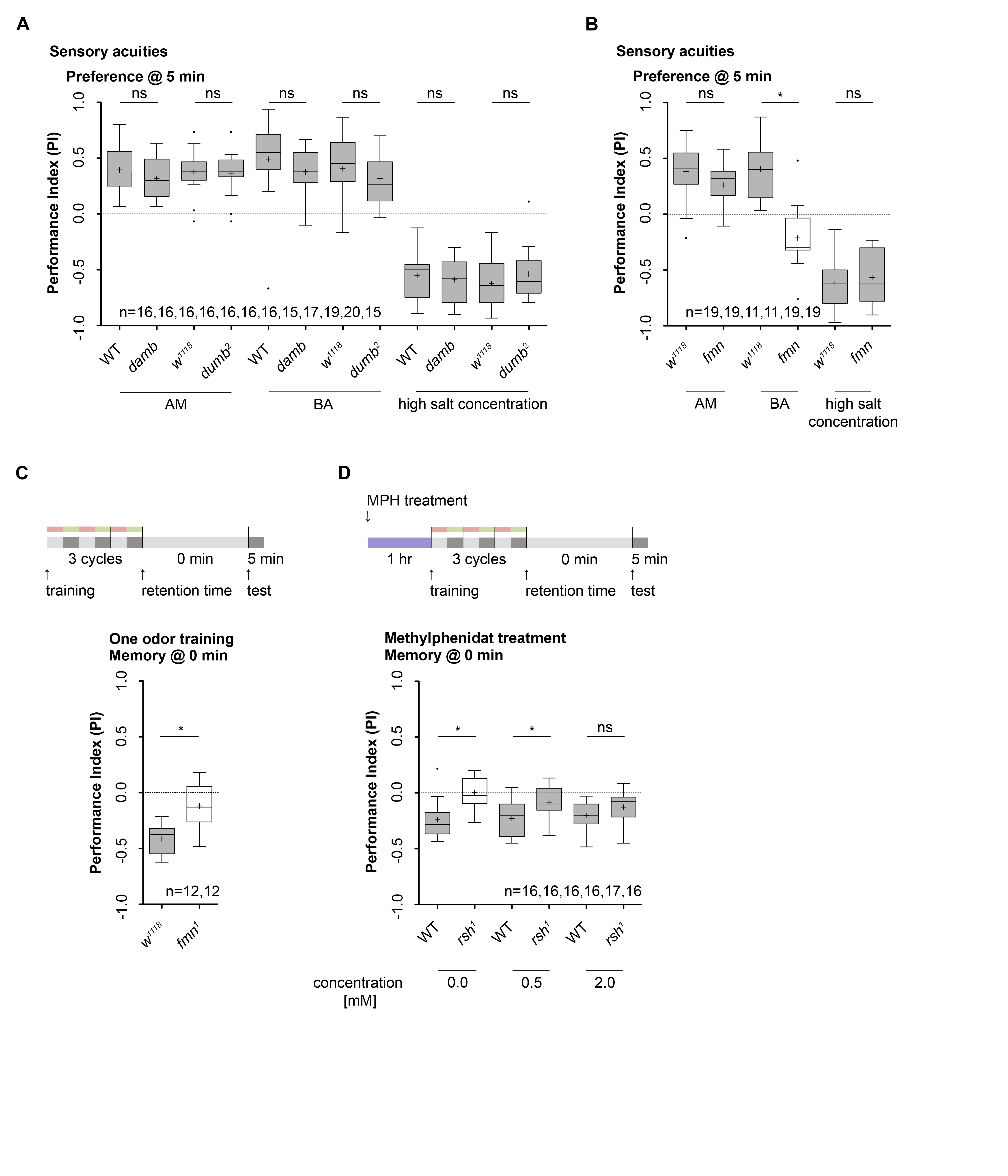

Supplement: S6 Fig — Training and methylphenidate (MPH) treatment protocols are shown at the top of each panel. A: Sensory acuity tests of the dopamine (DA) receptor mutants dumb2 and damb. Both receptors mutants perceived AM, BA and high salt stimuli comparable to controls (for AM: unpaired t test, p = 0.25 and p = 0.83, for BA: Mann-Whitney test, p = 0.07 and unpaired t test, p = 0.41, for high salt: unpaired t test, p = 0.57 and p = 0.28). B: Sensory acuity tests of the DAT mutant fumin (fmn). fmn mutant larvae showed no difference in their naïve responses to AM and high salt (Unpaired t test, p = 0.10 and p = 0.64, respectively). However, the naïve response to BA was impaired (Unpaired t test, p = 0.06). C: In line with the results for two odor conditioning, fmn mutant larvae using a one odor learning paradigm (to omit BA as a sensory stimulus) behaved significantly different compared to wild type control larvae (paired t test, p = 0.0003). They showed no aversive olfactory learning and/or memory (One sample t test, p = 0.06). D: Aversive olfactory memory in rsh1 and wild type control larvae after methylphenidate (MPH) treatment using different concentrations. Memory was tested directly after three cycle standard training. Aversive olfactory learning and memory was indistinguishable from random distribution for the rsh1 mutant without MPH application (One sample t test, p = 0.94). After application of 0.5 mM MPH rsh1 mutant larvae showed reduced learning and/or memory compared to wild type controls (One sample t test, p = 0.03, unpaired t test, p = 0.01). After application of 2.0 mM MPH rsh1 mutant larvae performed as wild type controls (Unpaired t test, p = 0.11). Sample size is n = 16 for each group if not indicated otherwise. Differences between groups are depicted above or below the respective box plots; ns indicates p≥0.05 and * p<0.05. Grey boxes indicate a memory performance above chance level (p<0.05), whereas white boxes indicate a memory performance at chance level (p≥0.05 [file pgen.1006378.s006.tif]

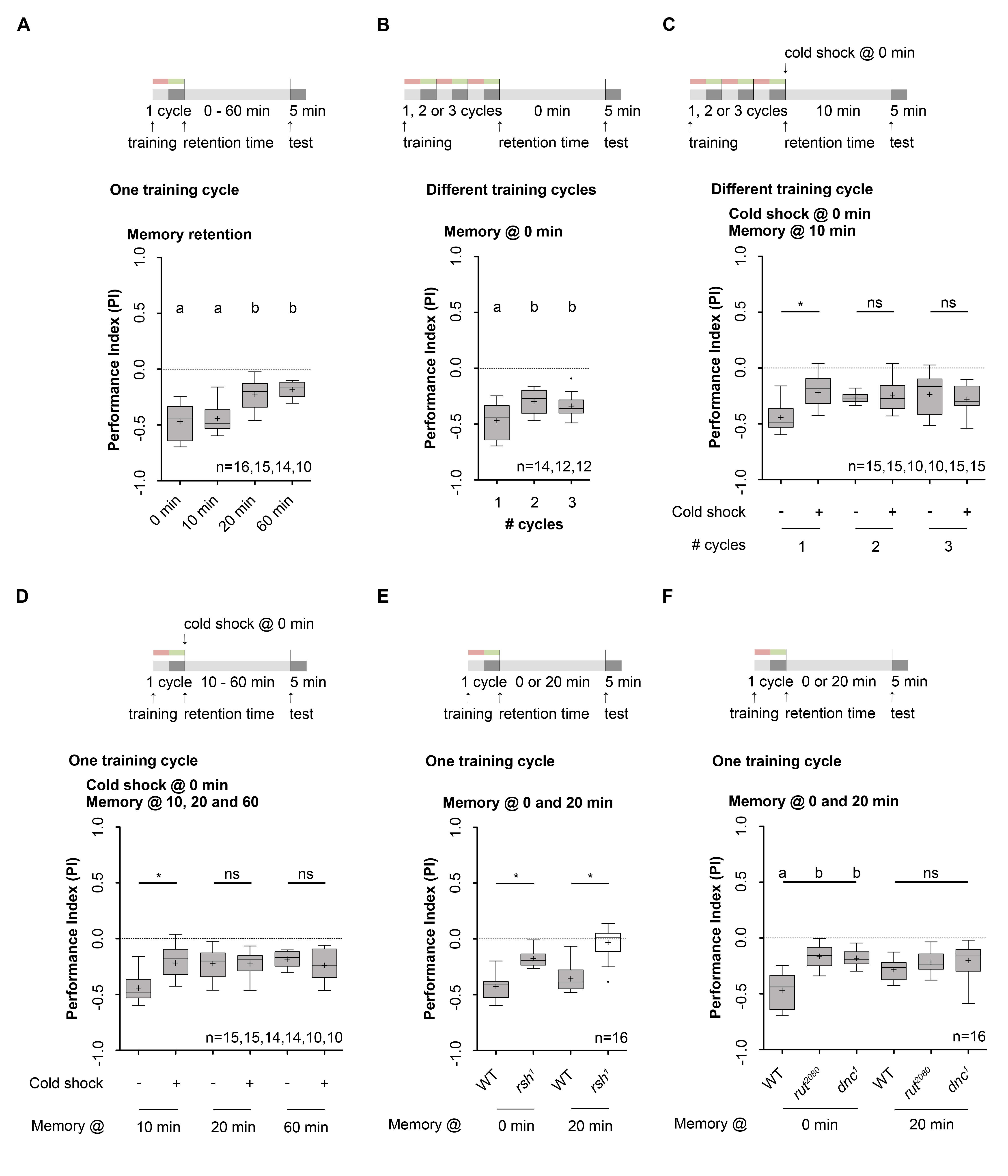

Supplement: S7 Fig — Training and cold shock treatment protocols are shown at the top of each panel. A: Aversive olfactory learning and/or memory using a one cycle training protocol was tested at four different retention times after conditioning (0, 10, 20 and 60 minutes). Statistical significant differences were revealed between the groups (One way ANOVA, p<0.0001). The performance indices measured immediately and 10 minutes after one cycle training were indistinguishable from each other (Tukey post hoc test, p = 0.945). Alike the performance indices measured 20 and 60 minutes after once cycle training showed no statistical significance differences (Tukey post hoc test, p = 0.874). However the performance indices measured 0 and 10 minutes after conditioning where at a higher level than the ones measured 20 and 60 minutes after conditioning when analyzed with Tukey post hoc test (p<0.0001 for 0 and 20, p<0.0001 for 0 and 60, p = 0.0001 for 10 and 20 and p<0.0001 for 10 and 60). B: Aversive olfactory learning and memory established with increasing training cycles (One cycle, two cycles and three cycles) revealed significant differences between one cycle training and two or three cycle training (One way ANOVA, p = 0.003; Tukey post hoc test p = 0.037, p = 0.033, respectively). For two and three cycle training no difference was detected (p = 0.705). C: Cold shock treatment directly after training partially impaired aversive olfactory learning and/or memory when tested 10 minutes after one cycle training. No difference was detected between cold shock treated and control larvae tested 10 minutes after two and three cycle training (Unpaired t-test, p = 0.0001 for one training cycle, p = 0.623 for two training cycles and p = 0.396 for three training cycles). D: Aversive olfactory learning and memory of cold shock treated larvae and control groups tested 10, 20 and 60 minutes after one cycle training. Only cold shocked treated larvae tested 10 minutes after one cycle training showed a significa [file pgen.1006378.s007.tif]
